# Induction of cardiac alternans in human iPS‐derived cardiomyocytes through β‐adrenergic receptor stimulation

**DOI:** 10.14814/phy2.70152

**Published:** 2024-12-23

**Authors:** Yuto Hinata, Daisuke Sasaki, Katsuhisa Matsuura, Tatsuya Shimizu

**Affiliations:** ^1^ Ogino Memorial Laboratory Nihon Kohden Corporation, TWIns Shinjuku‐ku Tokyo Japan; ^2^ Institute of Advanced Biomedical Engineering and Science TWIns, Tokyo Women's Medical University Shinjuku‐ku Tokyo Japan; ^3^ Department of Pharmacology Tokyo Women's Medical University Shinjuku‐ku Tokyo Japan

**Keywords:** calcium alternans, cardiac alternans, cardiac contractility, cell sheet technology, human‐induced pluripotent stem cell‐derived cardiomyocytes

## Abstract

Cardiac alternans (C‐ALT) is a phenomenon of alternating strong and weak contractions in the heart and is considered a risk factor for the development of heart failure and arrhythmias. However, no model has been reported that can induce C‐ALT in vitro using human cells, and the developmental mechanism of C‐ALT has not been studied using human cells. In this study, we successfully induced C‐ALT in vitro using human‐induced pluripotent stem cell‐derived cardiomyocytes (hiPSC‐CMs). By stimulating β‐adrenergic receptor with isoproterenol on hiPSC‐CMs cultured in atmospheric condition (with ~0.04% CO_2_), contractility and calcium transient were observed to alternately increase and decrease with each beat. In contrast, C‐ALT was not induced in hiPSC‐CMs cultured at 5% CO_2_ concentration. Since previous studies have linked C‐ALT to problems with calcium regulation in the sarcoplasmic reticulum (SR), we exposed hiPSC‐CMs to compounds that alter SR Ca^2+^ loading and analyzed their contractile responses. The results showed that exposure to verapamil, thapsigargin, and ryanodine either suppressed or eliminated C‐ALT. In contrast, omecamtiv mecarbil and blebbistatin, which alter contractility without SR Ca^2+^ loading, did not induce or suppress C‐ALT. These results suggest that C‐ALT in hiPSC‐CMs induced by isoproterenol may be due to abnormal regulation of the ryanodine receptor's opening and closing caused by excessive Ca^2+^ load in the SR from β‐adrenergic receptor stimulation.

## 1 INTRODUCTION

Cardiac alternans (C‐ALT) is a condition where the heart alternates between strong and weak contractions at a constant rate (Edwards & Blatter, 2014; Zhilin & Weiss, 2023). It is frequently detected in patients with chronic heart failure and is recognized as a risk factor for arrhythmias and sudden cardiac death (Kodama et al., 2001). In animal experiments, C‐ALT has been observed in the whole heart, isolated cardiac fragments, and primary cultured cells (Lab & Lee, 1990; Mcgaughey et al., 1985; Orchard et al., 1991). Experiments have also shown that high‐frequency electrical stimulation, acidosis, and β‐adrenergic receptor stimulation can induce C‐ALT (Florea & Blatter, 2012; Orchard et al., 1991). C‐ALT can be explained by two cellular mechanisms. One is voltage‐driven alternans, and the other is the calcium‐driven alternans (Jordan & Christini, 2007; Walker & Rosenbaum, 2003; Wan et al., 2012; Zhilin & Weiss, 2023). In voltage‐driven alternans, ion channels that form an action potential duration (APDs) do not recover in time for the next depolarization, resulting in alternating long and short APDs. Because membrane potential and Ca^2+^ homeostasis is coupled via Ca^2+^‐dependent ion currents, APD alternans may lead to Ca^2+^ alternans and ultimately to C‐ALT.

Calcium‐driven alternans is the mechanism directly caused by disturbances in Ca^2+^ homeostasis and problems with regulating intracellular Ca^2+^ in cardiomyocytes (Kulkarni et al., 2019; Shkryl et al., 2012; Wang, Myles, et al., 2014; Zhilin et al., 2016; Zhilin & Weiss, 2023).

Cardiac contraction in normal cardiomyocytes is triggered by Ca^2+^ entering through L‐type Ca^2+^ channels (LTCC) during depolarization, which then induces massive Ca^2+^ release from the sarcoplasmic reticulum (SR); Ca^2+^ released from the SR binds to myofilaments, leading to contraction. Ca^2+^ is then replenished in the SR by Ca^2+^ reuptake via the sarco/endoplasmic reticulum Ca^2+^‐ATPase (SERCA) pump; Ca^2+^ entering the cell via LTCC is pushed out of the cell via Na^+^–Ca^2+^ exchanger (NCX). A key aspect of understanding C‐ALT is that the strength of cardiac contraction is mainly controlled by the amount of Ca^2+^ released from the SR, which stores Ca^2+^. As expected, in cardiomyocytes with C‐ALT, the amount of Ca^2+^ released from the SR fluctuates with each beat.

In cardiomyocytes with C‐ALT, where alternating increases and decreases in Ca^2+^ release from the ryanodine receptor (RyR) occur, it has been confirmed that the amount of Ca^2+^ stored in the SR prior to contraction also fluctuates with each beat (Díaz et al., 2004). The amount of Ca^2+^ stored in the SR is mainly regulated by SERCA, which loads Ca^2+^ from the cytoplasm into the SR. Thus, SERCA activity can significantly affect C‐ALT development. Indeed, experiments have shown that alterations in SERCA expression in ventricular myocytes influence the development of C‐ALT (Xie et al., 2008).

During extremely high‐frequency pacing, it has been observed that the amount of Ca^2+^ released from the RyR alternately increases and decreases, causing C‐ALT, although the Ca^2+^ load of the SR remains constant during each beat (Picht et al., 2006). This phenomenon is reportedly caused by refractoriness, a RyR characteristic where the receptor needs time to reopen after closing (Rovetti et al., 2010; Shkryl et al., 2012; Zhilin et al., 2016). RyRs clustered on the SR membrane, known as Ca^2+^ release units (CRUs), fire individually and randomly in response to Ca^2+^ influx through LTCC. Ca^2+^ released from the SR by small‐scale fires triggers fires in surrounding CRUs, but as Ca^2+^ release from CRUs increases due to increased Ca^2+^ influx from the LTCC and increased Ca^2+^ storage in the SR, it spreads to more CRUs and fires them, leading to a cell‐wide Ca^2+^ fire, called calcium transient (CaT). At high beat frequencies, some CRUs that fire do not recover from refractoriness in time, resulting in an insufficient CRUs releasing Ca^2+^ from the SR during the next beat. As a result, CaT is smaller in one of the two consecutive beats, causing C‐ALT. Therefore, in C‐ALT cardiomyocytes, which are not caused by changes in Ca^2+^ stores in the SR, the recovery time from RyR refractoriness is closely related to the development of C‐ALT.

When investigating C‐ALT mechanisms in vitro, it is important to note that most of the studies reported to date have been conducted primarily in animals. This is because there is a risk of unknown confounding factors, such as differences in structure and drug response between animals and humans (Haghighi et al., 2003; Pang et al., 2019). In addition, the ethical issues surrounding animal experimentation have led to calls for developing alternative methods to reduce the number of animals used in research. Using human‐induced pluripotent stem cell‐derived cardiomyocytes (hiPSC‐CMs) to generate in vitro cardiac models is promising, as it avoids both species differences and ethical issues.

In this study, we successfully reproduced C‐ALT in vitro using hiPSC‐CMs. Stimulating β‐adrenergic receptors with isoproterenol in hiPSC‐CMs cultured under normal CO_2_ conditions (~0.04%), caused contractility and CaT to alternately increase and decrease with each beat. Furthermore, the study suggests that the C‐ALT mechanism was likely caused by an excessive increase in Ca^2+^ stored in the SR, which affected the open/close regulation of the RyR, and as a result, Ca^2+^ release from the SR was insufficient every other time. Interestingly, C‐ALT did not disappear even when the heart tissue's beating rate was reduced with ivabradine to a level considered sufficient for RyR to recover from the refractoriness. This result suggests that RyR refractoriness is influenced by factors other than time, or that other factors besides RyR refractoriness cause C‐ALT, which may provide new insights into C‐ALT mechanism.

## 2 METHODS

### 2.1 Production of hiPSC‐CMs

**FIGURE 1:**
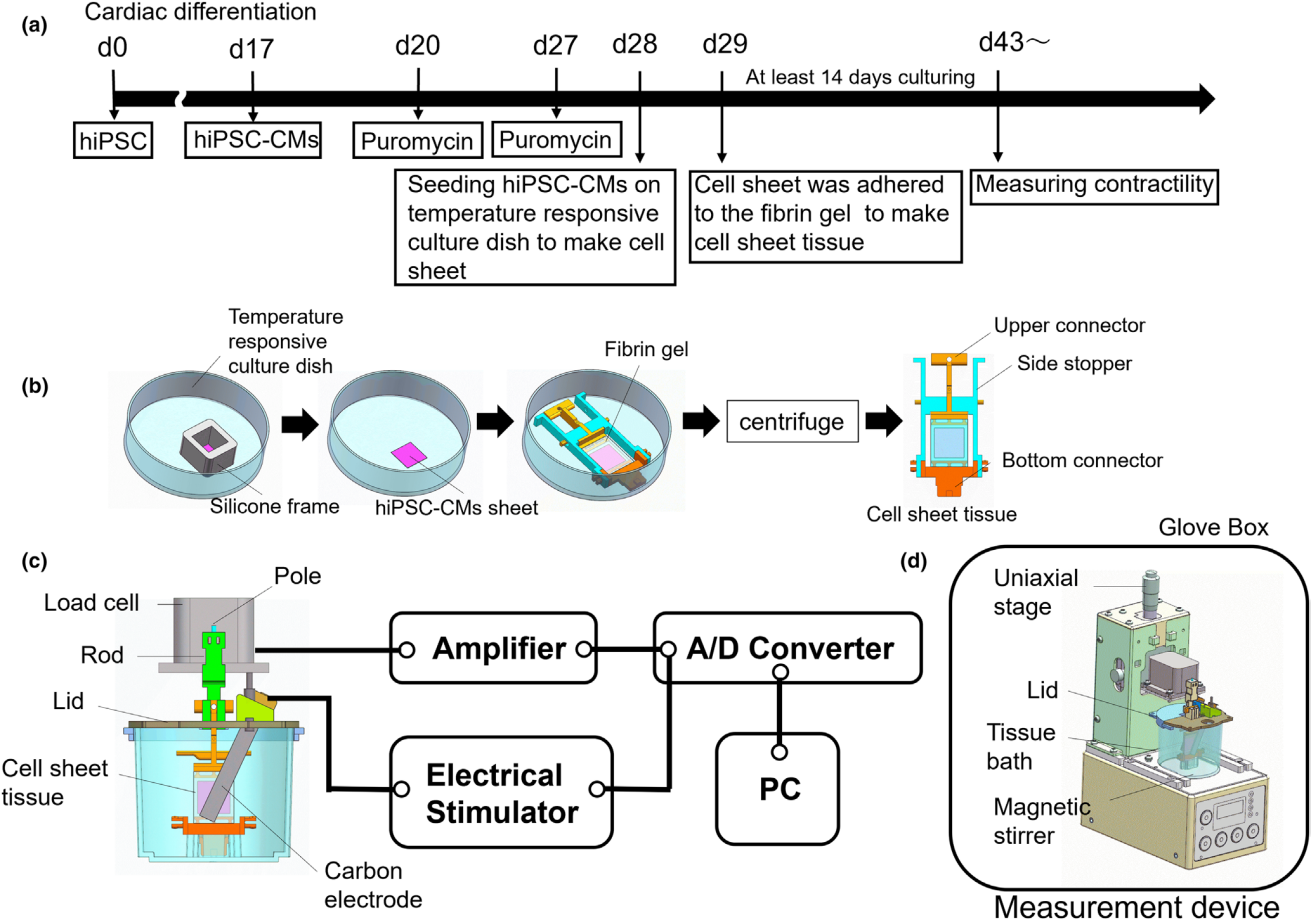
Fabrication of cardiac cell sheet tissue and measurement of contractility. (a) Time schedule of the experiments. (b) Fabrication of cardiac cell sheet tissues using fibrin gel and a temperature‐responsive culture dish. hiPSC‐CMs were seeded in a silicone frame on a temperature‐responsive culture dish to restrict the cell culture area and to prepare a square‐shaped cell sheet. Fibrin gel was prepared by mixing fibrinogen, thrombin, factor XIII, and CaCl_2_. Once the gel hardens, it was placed on the cell sheet in the temperature‐responsive culture dish and centrifuged to enable the cell sheet to adhere to the gel. (c) Schematic of the contraction force measurement system. The gel holder with the cell sheet tissue was attached to the bottom of the tissue bath containing the culture medium by inserting a bottom connector into a hole. After removing the side stopper, the upper connector was linked to the rod connected to the load cell, which measured the contraction force directly. The load cell was connected to an amplifier. The cell sheet tissue was stimulated by a pair of carbon electrodes positioned on both sides of the tissue as illustrated. (d) Schematic of the contraction force measurement device. The device had a magnetic stirrer to stir liquid in the tissue bath and was placed in a glove box maintained at 36.5°C ± 0.5°C.

The timeline for hiPSC‐CM generation is shown in Figure 1a. The hiPSC line 201B7, derived from a 36‐year‐old female, was purchased from RIKEN (Tsukuba, Japan) (Takahashi et al., 2007). The hiPSC expressing α‐myosin heavy chain and rex‐1 promoter–driven drug‐resistance genes were cultured on inactivated mouse embryonic fibroblasts (ReproCell, Yokohama, Japan) as described previously (Matsuura et al., 2016). The differentiation from hiPSCs to hiPSC‐CMs was performed using a stirred bioreactor system (Bio Jr.8; Able, Tokyo, Japan) following a published protocol (Matsuura et al., 2012). On day 17 after starting differentiation, cell aggregates were broken down using 0.05% trypsin/EDTA (Wako, Osaka, Japan). The cells were cultured in medium A, consisting of Dulbecco's modified Eagle's medium (043‐30085; Wako) with 10% (v/v) fetal bovine serum (FBS) and 1% (v/v) penicillin–streptomycin (Wako), and incubated at 37°C in a humidified condition with 5% CO_2_. On day 20, to eliminate non‐cardiomyocytes that lacked the puromycin‐resistant gene, the cultured cells were treated with 1.5 μg/mL puromycin (Thermo Fisher Scientific, Waltham, MA, USA) for 24 h. On day 21, the cultured cells were collected using 0.05% trypsin/EDTA and seeded onto gelatin‐coated culture dishes (190‐15805; Wako) at a density of 10.9–14.5 × 10^4^ cells/cm^2^. On day 27, the cultured cells were again treated with 1.5 μg/mL puromycin for 24 h, and, on day 28, viable cells were collected using 0.05% trypsin/EDTA. In this study, 20 differentiation batches of hiPSC‐CMs were produced and used in each experiment. The passage number of iPS cells used in this study is shown in Table S1.

### 2.2 Calcium imaging

To observe calcium dynamics in hiPSC‐CMs with C‐ALT, calcium imaging was performed. On day 28, hiPSC‐CMs were seeded at 15 × 10^4^ cells/cm^2^ in 96‐well plates coated with FBS (Nichirei Bioscience, Tokyo, Japan) and were cultured in medium A at 37°C in a humidified condition with 5% CO_2_ for 6 days. Culture medium was changed every 2 days. On day 34, the culture medium was changed to medium B. This medium included M199 medium (1235‐0039; Thermo Fisher Scientific) with 10% (v/v) FBS and 1% (v/v) penicillin–streptomycin (Wako). On day 35, to investigate the intracellular calcium transients, hiPSC‐CMs were loaded with Ca^2+^ sensitive dye (EarlyTox Cardiotoxicity Kit, R8210; MOLECULAR DEVICES, San Jose, CA, USA), diluted to 20% in culture medium B, and the cells were incubated at 37°C in a humidified atmosphere for 2 h. To stop the beating of hiPSC‐CMs, 25 μM of para‐aminoblebbistatin (ax494682; Axol Bioscience, Cambridge, UK) diluted in DMSO was exposed 20 min before observation. After 2 h of cultivation with the Ca^2+^ sensitive dye, calcium transients were recorded using fluorescence digital microscopy (BZ‐X810; Keyence, Osaka, Japan) with GFP filter (Keyence OP‐87763; Ex: 470/40 nm, Em: 525/50 nm) before and after exposure to 1 μM isoproterenol. Fluorescence emission was viewed with a 2X objective lens (Plan Apochromat; NA. 0.10). Time‐lapse images were acquired at 50‐ms intervals for 20 s (Movies S1 and S2). Two different differentiated batches were used in this experiment; one well was defined as *n* = 1. When observing calcium dynamics in the cardiac cell sheet tissues, a confocal quantitative image cytometer was used (CQ1; Yokogawa Electric Corporation, Tokyo, Japan). Ca^2+^ sensitive dye was excited at 488 nm using solid‐state laser, and fluorescence emission was viewed through a dry objective lens (UPLSAPO20X; NA. 0.75; Olympus). Time‐lapse images were acquired at 50‐ms intervals for 30 s (Movies S3 and S4).

### 2.3 Cardiac cell sheet tissue preparation

Cardiac cell sheet tissues were manufactured using a previously reported method with some modifications (Haraguchi et al., 2018; Hinata et al., 2022; Sasaki et al., 2018). First, a silicone frame was placed on a temperature‐responsive culture dish (UpCell, CS3006; CellSeed, Tokyo, Japan) to define the cell culture area and was coated with FBS and incubated for 1–2 days. Second, hiPSC‐CMs were seeded at 3 × 10^5^ cells/cm^2^ within the silicone frame and cultured in medium A at 37°C in a humidified condition with 5% CO_2_ for 24 h. A fibrin gel was prepared by mixing fibrinogen (F8630; Sigma‐Aldrich, St. Louis, MO, USA), thrombin (T4648; Sigma‐Aldrich), factor XIII (731119941; CSL Behring, King of Prussia, PA, USA), and CaCl_2_ (Kishida Chemical, Osaka, Japan) solution. After removing the medium and silicone frame, the fibrin gel was placed on the square‐shaped cardiac cell sheet in the temperature‐responsive culture dish (Figure 1b). To help the cardiac cell sheet stick to the fibrin gel, the dish was centrifuged at 200 × *g* for 5 min and then incubated for 60 min in an incubator at 37°C with 5% CO_2_. To detach the cardiac cell sheet from the culture dish, the dish was placed in another incubator at 20°C for 90 min with 5% CO_2_. Subsequently, to stop the cardiomyocytes from beating, the gel holder with the fibrin gel was lifted from the dish and placed in another dish filled with medium C, which contained medium A with 2 mg/mL aminocaproic acid (010‐09641; Fujifilm Wako Pure Chemical Corporation, Osaka, Japan) and 40 mM KCl (Kishida Chemical). The cell sheet tissue was cultured for 24 h in an incubator with 5% CO_2_ at 37°C. Thereafter, medium C was replaced with medium A with 2 mg/mL aminocaproic acid, and the cell sheet tissue was cultured for at least 10 days before measuring contractility.

### 2.4 Preparation of culture medium for measuring contractility

The culture medium used in measuring contractility in this study is shown in Table 1. Conditions 1, 2, and 3 were prepared based on M199 Hanks' salts medium (12350‐039; Thermo Fisher Scientific), while condition 4 was prepared based on M199 Earle's salts medium (11150‐059; Thermo Fisher Scientific). Each medium contained 10% (v/v) FBS, 1% (v/v) penicillin–streptomycin, and 2 mg/mL aminocaproic acid. Condition 3 was adjusted to pH 6.9 by adding hydrochloric acid (HCl) while measuring with a pH meter (F‐72; HORIBA, Kyoto, Japan) at 25°C in atmospheric condition.

**TABLE 1:** Culture medium and environment for measuring contractility of cardiac cell sheet tissues.

	Medium	Usage environment	Model No.	pH	
Condition 1	M199 Hanks' salts	CO_2_ 5%	12350‐039	6.9	
Condition 2	M199 Hanks' salts	Atmosphere	12350‐039	7.2	
Condition 3	M199 Hanks' salts	Atmosphere	12350‐039	6.9	pH adjusted with HCl
Condition 4	M199 Earles' salts	CO_2_ 5%	11150‐059	7.3	

### 2.5 Contraction force measurement

Contraction force was measured using a modified version of a previously reported method (Hinata et al., 2022). Figure 1c illustrates how the contraction force measurement system is set up (Nihon Kohden, Tokyo, Japan). The contraction force from the cell sheet tissue was directly measured using a load cell (LVS‐10GA; Kyowa Electronic Instruments, Tokyo, Japan) in the measurement device (Figure 1d). The cell sheet tissue was vertically placed in a tissue bath with 50 mL of each culture medium listed in Table 1. The gel holder was attached to the bottom of the tissue bath by inserting a connector into a hole in the bath. The upper connector was tightly connected to the load cell through a rod and pole (Figure 1c). The height of the load cell was adjusted using a uniaxial stage. The contraction force was measured in a glove box to keep the culture environment at 36.5°C ± 0.5°C. CO_2_ concentration was set to 5% or adjusted as needed based on the experiment. A magnetic stirrer was used to keep the temperature and compound concentration in the medium consistent. The load cell was connected to a strain amplifier (PP‐101H; Nihon Kohden), and the contraction force was recorded on LabChart (ADInstruments, Bella Vista, Australia)‐installed PC via an A/D converter (DC‐300H; Nihon Kohden). The sampling frequency was set to 100 Hz. Electrical pacing of the cell sheet tissue by field stimulation was performed using carbon plate electrodes positioned as shown in Figure 1c. A biphasic pacing pulse (6–10 V, 2.5‐ms pulse duration) was applied at 1.5 Hz using an electrical stimulator (SEN‐3401; Nihon Kohden).

### 2.6 Compound tests

Compound tests were conducted using cardiac cell sheet tissues with contractility measured for at least 1 day under each condition listed in Table 1 after incubation for at least 10 days. For pacing condition samples, electrical stimulation was performed at 1.5 Hz for at least 1 h before the compound test. The culture medium was not changed from the beginning of the contraction force measurement until the end of the compound tests. Isoproterenol (I0260; Tokyo Chemical Industry, Tokyo, Japan), propranolol (163‐24501; Fujifilm Wako Pure Chemical Corporation), verapamil (222‐00781; Fujifilm Wako Pure Chemical Corporation), ryanodine (ab120083; Abcam, Cambridge, UK), thapsigargin (10522; Cayman Chemical, MI, USA), omecamtiv mecarbil (CS‐0460; Chemscene LCC, NJ, USA), ivabradine (098–06921; Fujifilm Wako Pure Chemical Corporation), and blebbistatin (ax494682; Axol Bioscience) were dissolved in DMSO (031‐24051; Fujifilm Wako Pure Chemical Corporation) at concentrations 1000 times higher than those of the exposure test; the compound solutions were added to the medium in the tissue bath and diluted appropriately. The effects of 1000 nM isoproterenol, 1000 nM propranolol, 300 nM verapamil, 30 nM ryanodine, 1000 nM thapsigargin, 1000 nM omecamtiv mecarbil, 300 nM ivabradine, and 300 nM blebbistatin on cardiac contractility were evaluated, respectively. The compound test concentration was determined based on the studies by Mannhardt et al. (2016); Mannhardt et al. (2017); Mannhardt et al. (2020); Saleem et al. (2020); Arai et al. (2020). For verapamil and ryanodine, the concentration was set at a level that did not stop the beating, since higher concentrations caused contraction arrest in this study (data not shown). Isoproterenol was exposed to the cardiac cell sheet tissue for 1 h during contraction force measurements in each culture environment listed in Table 1. In the isoproterenol exposure test, different cardiac cell sheet tissues were used in each condition. Some of the tissues in which C‐ALT was induced by the isoproterenol exposure test were also used in the propranolol, verapamil, ryanodine, thapsigargin, and blebbistatin exposure test described later. Detailed information on the number of tissues used in each compound exposure test can be found in Table S1. Omecamtiv mecarbil was exposed for 30 min to the cardiac cell sheet tissue cultured under condition 2 as described in Table 1. Propranolol, verapamil, ryanodine, thapsigargin, and blebbistatin were each exposed for 30 min to the C‐ALT‐induced cardiac cell sheet tissue cultured under condition 2 at 1.5 Hz stimulation, respectively. Isoproterenol, propranolol, and ivabradine were exposed to cardiac cell sheet tissue under spontaneous beating condition for 30 min. In the propranolol and ivabradine exposure test under spontaneous beating conditions, the C‐ALT‐induced cardiac cell sheet tissues used in the isoproterenol exposure test were used. In this study, cardiac cell sheet tissues exposed to any compound except isoproterenol were not used for any other compound exposure test. The mechanism of action, exposure concentrations, and electrical stimulation conditions for each compound are listed in Tables 2 and 3. The passage number of iPS cells and differentiation batch of hiPSC‐CMs used to generate cardiac cell sheet tissues in each study are listed in Table S1.

**TABLE 2:** Experimental condition and observed responses in this study using cardiac cell sheet tissue under atmospheric condition.

Drug	Mode of action	Test condition	Conc. (nM)	Beat rate	Force	C‐ALT
Isoproterenol	β‐Adrenergic receptor agonist	Pacing (1.5 Hz)	1000	–	↑	★
		Spontaneous		↑	→	★
Omecamtiv mecarbil	Myosin activator	Pacing (1.5 Hz)	1000	–	↑	☆

*Note*: ★: induced; ☆: not induced; ↑: positive effects; →: not changed.

**TABLE 3:** Experimental condition and observed responses of cardiac cell sheet tissues with C‐ALT exposed to each compound.

Drug	Mode of action	Test condition	Conc. (nM)	Beat rate	Force	C‐ALT
Propranolol	β‐Adrenergic receptor antagonist	Pacing (1.5 Hz)	1000	–	↓	◆◆
		Spontaneous		↓	↓	◆◆
Verapamil	L‐type Ca^2+^ channel blocker	Pacing (1.5 Hz)	300	–	↓	◆
Thapsigargin	SERCA inhibitor	Pacing (1.5 Hz)	1000	–	↓	◆◆
Ryanodine	Ryanodine receptor inhibitor	Pacing (1.5 Hz)	30	–	↓	◆◆
Blebbistatin	Myosin inhibitor	Pacing (1.5 Hz)	300	–	↓	◇
Ivabradine	HCN channel inhibitor	Spontaneous	300	↓	↑	◆

*Note*: ◆◆: disappeared; ◆: suppressed; ◇: not disappeared or suppressed; ↑: positive effects ↓: negative effects. “Disappeared” was defined as one or more samples with the S/L ratio of 0.99 or greater after each compound exposure. “Suppressed” was defined as an increase in the S/L ratio, but less than 0.99, after each compound exposure.

### 2.7 Contraction waveform parameters

To quantitatively characterize the contraction force waveform, two parameters, which were analyzed by LabChart, were defined based on a previously reported method (Hinata et al., 2022): contraction amplitude (CA), wave height value (unit: mN); and relaxation time (RT), time taken for the tension to fall from 100% to 10% of the wave height value (unit: s). The peak of contraction was detected using the Peak Analysis module (v1.5.1) for LabChart with the appropriate settings, and the two parameters were calculated for each detected peak.

### 2.8 Quantitation of C‐ALT and contraction force

Since C‐ALT was characterized by alternating repetition of large (L) and small (S) wave heights, C‐ALT induction was quantified by the ratio of contraction force between two consecutive contraction waveforms (S/L ratio), based on Florea and Blatter (2012). Initially, six pairs of contraction waveforms were extracted from 12 consecutive beats. The ratio of the wave heights was calculated for each of the six pairs of waveforms, and the average was set as the S/L ratio. Under normal conditions, the contraction waveform has a continuous shape with a constant wave height, and alternately repeating large (L) and small (S) wave heights under C‐ALT induction. Therefore, the S/L ratio ranges from 1 to 0. A value of 1 means no C‐ALT, while a value less than 1 indicates that C‐ALT is significantly induced. To evaluate the effects of the compounds on contraction force, waveform data from 12 consecutive beats were collected. Six alternate waveform data (large waveform) from these beats were selected for analysis and averaged. The CA values presented in this study were normalized to the values obtained before exposure to the compounds.

### 2.9 RNA extraction and quantitative RT‐PCR

RNA was extracted from each of three different differentiation batches of hiPSC‐CMs, and quantitative RT‐PCR was performed. On day 28, treated with the second puromycin for 24 h, total RNA from hiPSC‐CMs was isolated using RNeasy Mini Kit (74106; Qiagen, Hilden, Germany) according to the manufacturer's manual. Total RNA of primary adult heart was purchased from Takara Bio USA, Inc. (Human Heart Total RNA, 636532; lot 2002947A). RNA concentrations were measured using NanoDrop ONE Spectrophotometer (Thermo Fisher Scientific). Complementary DNA was synthesized from the purified total RNA using a High‐Capacity cDNA Reverse Transcription Kit with RNase Inhibitor (4374966; Thermo Fisher Scientific). Quantitative RT‐PCR was performed using an Applied BioSystems® ViiA™ 7 RT‐PCR system (Thermo Fisher Scientific) in accordance with the manufacturer's manual. Gene expression levels were analyzed using TaqMan gene expression assays (Thermo Fisher Scientific). Each PCR reaction mixture contained 10 μL of TaqMan Fast Advanced Master Mix (4444557; Thermo Fisher Scientific), 1 μL of diluted cDNA reaction mixture (corresponding to 100 ng RNA starting amount), and 900 nM of each primer in a total reaction volume of 20 μL. Taqman probes used are described in Table 4. The data were analyzed using the △CT method with GAPDH set as the reference gene. Each PCR reaction was performed in triplicate.

**TABLE 4:** PCR primer information.

Gene name	Thermo fisher scientific No.
Glyceraldehyde‐3‐phosphate dehydrogenase (GAPDH)	Hs99999905_m1
Ryanodine receptor 2 (cardiac) (RYR2)	Hs00181461_m1
Calsequestrin 2 (CASQ2)	Hs00154286_m1
Histidine rich calcium binding protein (HRC)	Hs05054575_s1
Triadin (TRDN)	Hs00952568_m1

### 2.10 Data and statistical analysis

In compound test, the number of tissue samples was defined as *n*. A paired two‐tailed *t*‐test was used to compare the data before and after compound exposure, with a *p* value of <0.05 indicating statistical significance. Statistical analyses were performed using R software (version 4.3.0; R Foundation for Statistical Computing, Vienna, Austria) and Microsoft Excel (Microsoft, Redmond, WA, USA).

## 3 RESULTS

### 3.1 Inducing C‐ALT using cardiac cell sheet tissue by β‐adrenergic receptor stimulation

**FIGURE 2:**
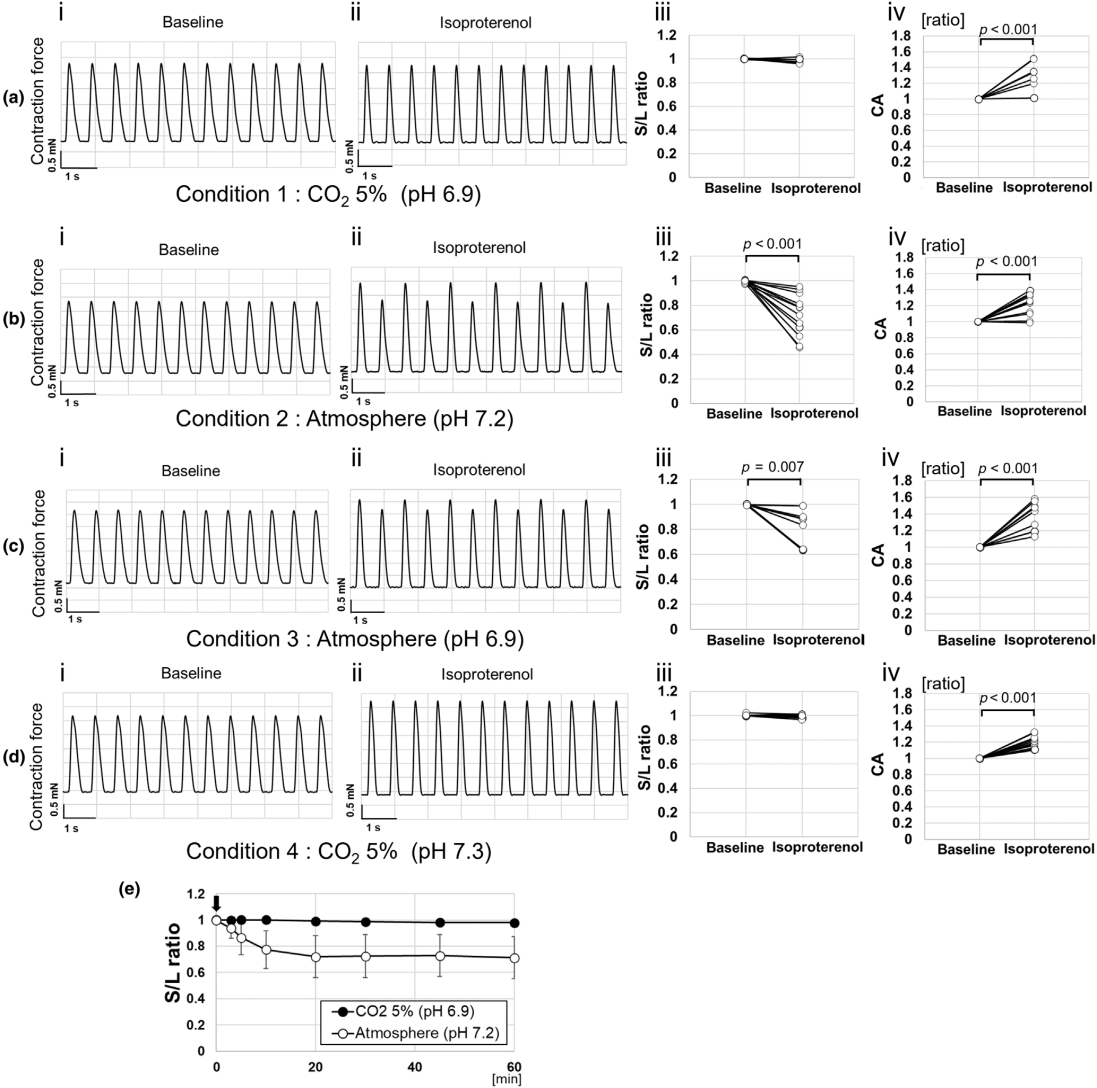
Effects of CO_2_ concentration and pH on isoproterenol response of cardiac cell sheet tissues. (a–d) Typical contraction waveform, S/L ratio (each sample data), and CA (contraction amplitude) value (each sample data) of cardiac cell sheet tissues before (baseline) and 30 min after isoproterenol exposure. (a) The data of cardiac cell sheet tissues cultured in Hanks' salt medium at 5% CO_2_ concentration (S/L ratio [baseline]; mean ± S.D. = 1.00 ± 0.00, S/L ratio [isoproterenol]; 0.99 ± 0.02, CA [isoproterenol]; 1.32 ± 0.16, *n* = 8). (b) The data of cardiac cell sheet tissues cultured in Hanks' salt medium in an atmosphere (S/L ratio [baseline]; mean ± S.D. = 1.00 ± 0.01, S/L ratio [isoproterenol]; 0.72 ± 0.16, CA [isoproterenol]; 1.22 ± 0.13, *n* = 13). (c) The data of cardiac cell sheet tissues cultured in Hanks' salt medium in an atmosphere (S/L ratio [baseline]; mean ± S.D. = 1.00 ± 0.00, S/L ratio [isoproterenol]; 0.82 ± 0.15, CA [isoproterenol]; 1.37 ± 0.17, *n* = 9). (d) The data of cardiac cell sheet tissues cultured in Earle's salt medium at 5% CO_2_ concentration (S/L ratio [baseline]; mean ± S.D. = 1.00 ± 0.01, S/L ratio [isoproterenol]; 0.99 ± 0.01, CA [isoproterenol]; 1.20 ± 0.07, *n* = 12). CA was normalized to baseline. (e) Time course of S/L ratio when exposed to isoproterenol under atmosphere (CO_2_ concentration was ~0.04%) and 5% CO_2_ concentration using M199 Hanks' salt medium. One micromolar of isoproterenol was exposed at 0 min (indicated by arrow).

Spontaneous beating was visually observed in cardiac cell sheet tissues incubated at 37°C in a humidified condition with 5% CO_2_ for more than 10 days. In addition, the contraction force of the cardiac cell sheet tissues was measured under 1.5‐Hz electrical pacing with the media from conditions 1, 2, 3, and 4 listed in Table 1. As a result, continuous contraction waveforms with uniform heights were obtained in all conditions (Figure 2a–d).

To observe the culture conditions that induce C‐ALT, isoproterenol was exposed to the cardiac cell sheet tissue, and contraction force was measured under each condition listed in Table 1. Before exposure, the S/L ratio was 1.00 ± 0.00 and after 60 min of exposure it was 0.98 ± 0.03 in condition 1, showing little change (*p* = 0.060, Figure 2e). On the contrary, the S/L ratio before exposure was 1.00 ± 0.01, but it gradually decreased after exposure, reaching 0.71 ± 0.16 after 60 min in condition 2 (*p* < 0.001, Figure 2e).

The S/L ratio reached a steady state at 30 min after isoproterenol exposure as shown in Figure 2e. Representative waveforms, S/L ratio, and CA under each condition at 30 min after isoproterenol exposure are shown in Figure 2a–d. In condition 1, the S/L ratio did not change significantly from 1.00 ± 0.00 before isoproterenol exposure to 0.99 ± 0.02 after isoproterenol exposure (*p* = 0.076, Figure 2a). The CA value increased to 1.32 ± 0.16 based on the contraction force before isoproterenol was added (*p* < 0.001, Figure 2a). In condition 2, the S/L ratio decreased from 1.00 ± 0.01 before isoproterenol exposure to 0.72 ± 0.16 after exposure. The CA value after isoproterenol exposure was 1.22 ± 0.13 based on the contraction force before exposure, with some samples showing an increase and others showing no change. (*p* < 0.001, Figure 2b). In condition 3, the S/L ratio decreased from 1.00 ± 0.00 before isoproterenol exposure to 0.82 ± 0.15 after exposure, with some samples showing C‐ALT and others not (*p* = 0.007, Figure 2c). Contraction force increased to 1.37 ± 0.17 compared to before isoproterenol exposure (*p* < 0.001). In condition 4, the S/L ratio did not change significantly from 1.00 ± 0.01 before isoproterenol exposure to 0.99 ± 0.01 after isoproterenol exposure (*p* = 0.102). Contraction force increased to 1.20 ± 0.07 compared to before isoproterenol exposure (*p* < 0.001, Figure 2d).

These results indicate that C‐ALT can be reproducibly induced in hiPSC‐CMs cultured under atmospheric conditions when exposed to isoproterenol. Therefore, condition 2 was used to induce C‐ALT in the following experiments.

### 3.2 Calcium dynamics in hiPSC‐CMs with C‐ALT

**FIGURE 3:**
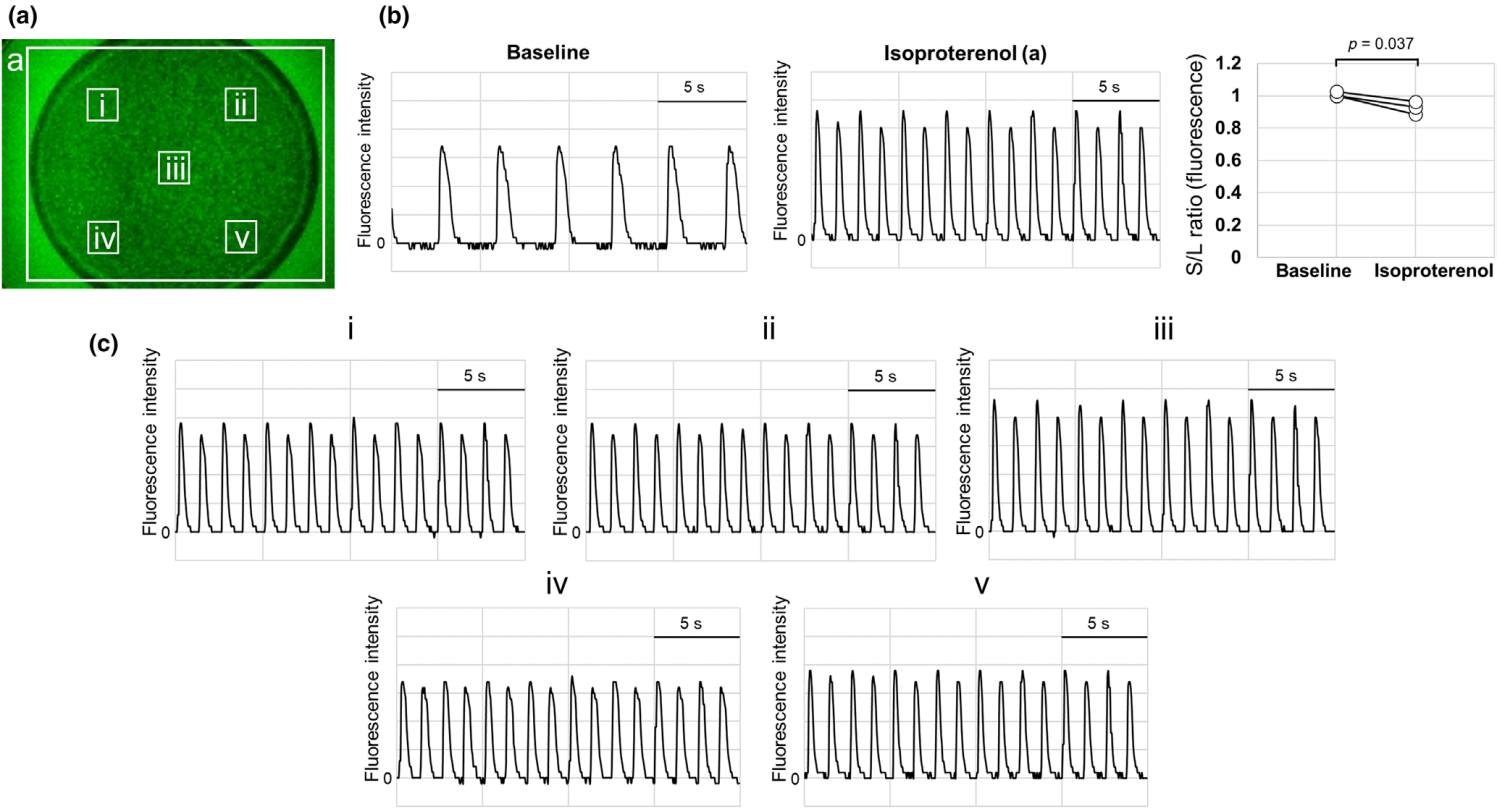
Calcium transient of hiPSC‐CM exposed to isoproterenol under atmospheric condition. (a) Fluorescent image of hiPSC‐CMs cultured on a 96‐well plate. White squares indicate the ROIs used to quantify fluorescence intensity. Movie S1 shows the hiPSC‐CMs before one micromolar of isoproterenol exposure, and Movie S2 shows hiPSC‐CMs 15 min after isoproterenol exposure. (b) Fluorescence intensity waveform quantified from region a fluorescence images before and after isoproterenol exposure. The graph on the right shows the S/L ratio (each sample data) calculated from the fluorescence intensity waveform (S/L ratio [baseline]; mean ± S.D. = 1.01 ± 0.02, S/L ratio [isoproterenol]; 0.93 ± 0.04, *n* = 3). (c) Fluorescence intensity waveform quantified from five regions (i, ii, iii, iv, and v) in Figure 3a.

The calcium dynamics of C‐ALT in hiPSC‐CMs under spontaneous beating conditions were observed by calcium imaging. To induce C‐ALT, hiPSC‐CMs cultured in a 96‐well plate were exposed to isoproterenol under atmospheric conditions. As a result, the S/L ratio (fluorescence) decreased from 1.01 ± 0.02 to 0.93 ± 0.04 (*p* = 0.037, Figure 3a,b). Movies S1 and S2 show the cells before and after isoproterenol exposure. In addition, fluorescence intensity was quantified from five ROIs on the 96‐well plate, and it increased and decreased with each beat in all ROIs (Figure 3c). As shown in Figure 3b,c, both the large and small waveforms dropped to the same baseline fluorescence intensity each time. Further observation of the cardiac cell sheet tissue with C‐ALT at higher magnification showed that the fluorescence intensity alternated up and down within a single cell (before and after isoproterenol exposure are shown in Movies S3 and S4). These results suggest that the C‐ALT observed in this study using hiPSC‐CMs occurs in individual cells, rather than a partial lack of contractile movement in the cardiac cell sheet tissue.

### 3.3 Effects of positive inotropes on cardiac cell sheet tissues under atmospheric condition

**FIGURE 4:**
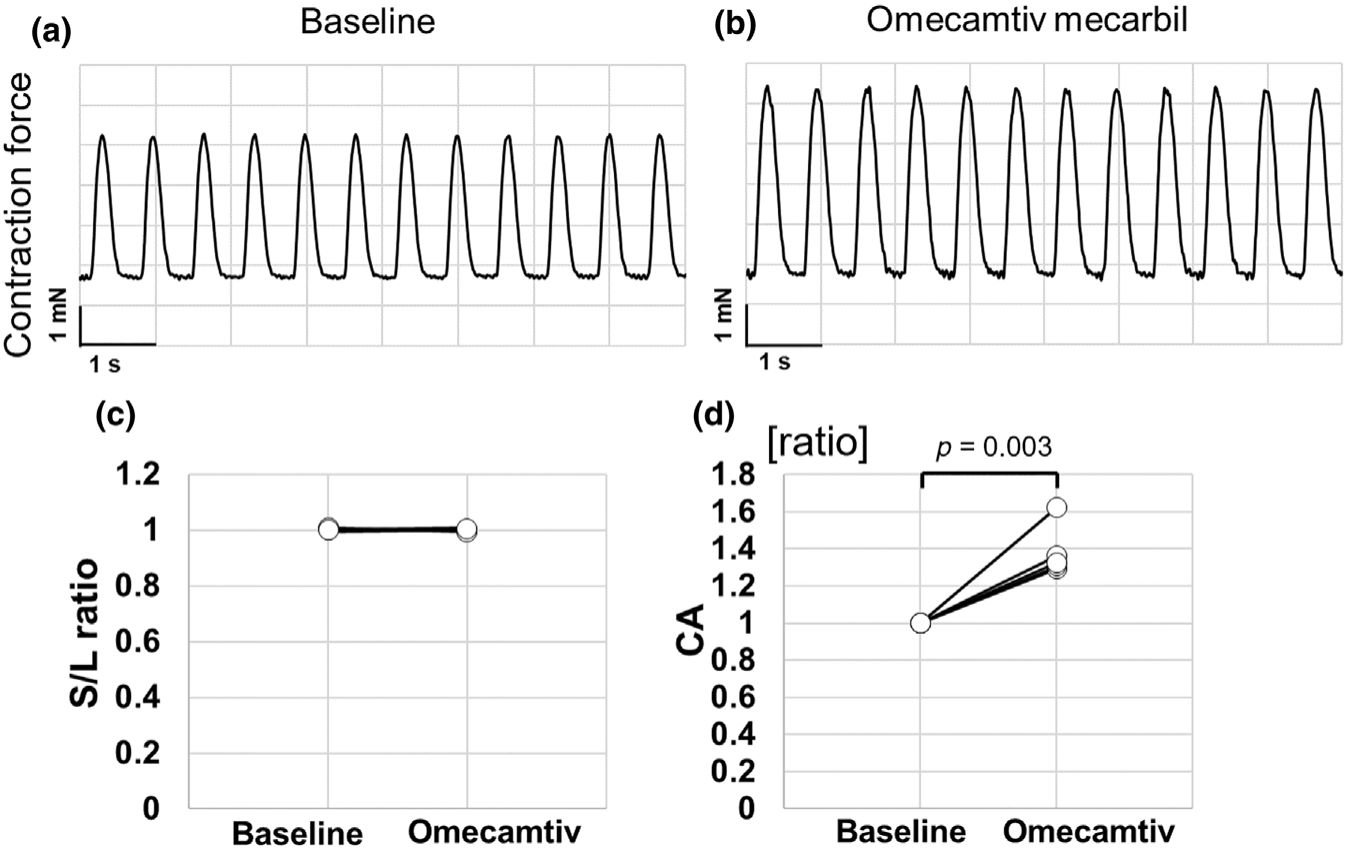
Effects of omecamtiv mecarbil on contractility of cardiac cell sheet tissues cultured under atmospheric condition. (a) and (b) show typical contraction waveform of cardiac cell sheet tissue, cultured under atmospheric condition using Medium 199 Hanks' salt medium, before (baseline) and after 30 min to 1000 nM omecamtiv mecarbil. (c) and (d) show the S/L ratio (each sample data) and CA (contraction amplitude) value (each sample data) (S/L ratio [baseline]; mean ± S.D. = 1.00 ± 0.01, S/L ratio [isoproterenol]; 1.00 ± 0.01, CA [isoproterenol]; 1.38 ± 0.14, *n* = 5). CA was normalized to baseline.

Isoproterenol, which induced C‐ALT in this study, stimulates β‐adrenergic receptors, thereby altering the activity of channel proteins involved in calcium dynamics, such as LTCC. This results in increased intracellular Ca^2+^ distribution and increased contraction force. Therefore, to determine if increased intracellular Ca^2+^ is involved in the induction of C‐ALT, omecamtiv mecarbil, which increases myosin Ca^2+^ sensitivity and increases contractile force without changing intracellular Ca^2+^ distribution, was tested under 1.5‐Hz pacing (Table 2). As a result, omecamtiv mecarbil exposure increased the CA value to 1.38 ± 0.14 (*p* = 0.003), while the S/L ratio remained unchanged at 1.00 ± 0.01 before exposure (*p* = 0.668, Figure 4).

### 3.4 Effects of compounds on C‐ALT in cardiac cell sheet tissues under electrical pacing

**FIGURE 5:**
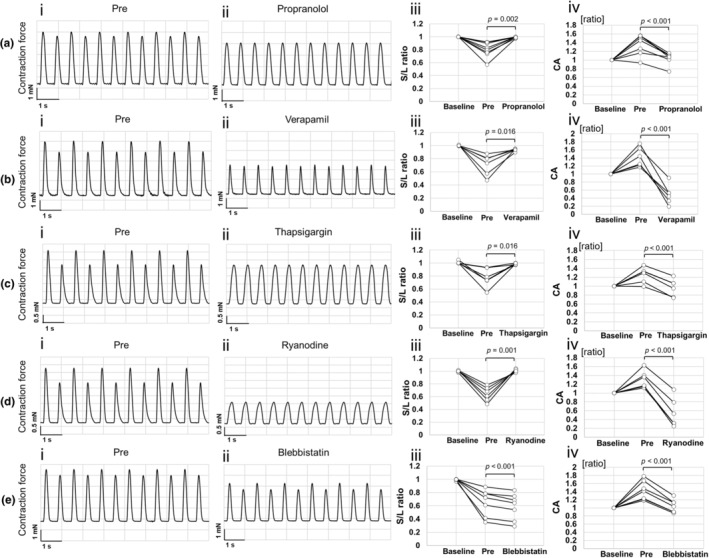
Effects of compounds on contractility of cardiac cell sheet tissues with C‐ALT cultured under atmospheric condition. (a–e) The typical contraction waveform, S/L ratio (each sample data), and CA (contraction amplitude) value (each sample data) of cardiac cell sheet tissue, cultured under atmospheric condition using Medium 199 Hanks' salt medium, before and after 30 min exposure to 1000 nM propranolol (S/L ratio [pre]; mean ± S.D. = 0.80 ± 0.12, S/L ratio [propranolol]; 0.99 ± 0.01, CA [pre]; 1.38 ± 0.23, CA [propranolol]; 1.05 ± 0.13, *n* = 8), 300 nM verapamil (S/L ratio [pre]; mean ± S.D. = 0.71 ± 0.16, S/L ratio [verapamil]; 0.94 ± 0.02, CA [pre]; 1.41 ± 0.24, CA [verapamil]; 0.49 ± 0.24, *n* = 6), 1000 nM thapsigargin (S/L ratio [pre]; mean ± S.D. = 0.78 ± 0.15, S/L ratio [thapsigargin]; 0.99 ± 0.01, CA [pre]; 1.25 ± 0.18, CA [thapsigargin]; 0.97 ± 0.19, *n* = 6), 30 nM ryanodine (S/L ratio [pre]; mean ± S.D. = 0.64 ± 0.11, S/L ratio [ryanodine]; 1.01 ± 0.02, CA [pre]; 1.30 ± 0.20, CA [ryanodine]; 0.55 ± 0.33, *n* = 6), and 300 nM blebbistatin (S/L ratio [pre]; mean ± S.D. = 0.65 ± 0.20, S/L ratio [blebbistatin]; 0.59 ± 0.20, CA [pre]; 1.42 ± 0.23, CA [blebbistatin]; 1.04 ± 0.16, *n* = 7), respectively. “Baseline” indicates no exposure to any compounds. “Pre” indicates that C‐ALT is induced by exposure to 1000 nM isoproterenol. CA was normalized to baseline.

To determine the changes in calcium kinetics that trigger C‐ALT, several compounds targeting different proteins involved in calcium kinetics were exposed to C‐ALT‐induced cardiac cell sheet tissues under 1.5‐Hz electrical pacing, and their effects on contractility were assessed (Table 3). Exposure to the β‐adrenergic receptor blocker propranolol increased the S/L ratio from 0.80 ± 0.12 to 0.99 ± 0.01 (*p* = 0.002) and decreased the CA value from 1.38 ± 0.23 to 1.05 ± 0.13 (*p* < 0.001, Figure 5a). Exposure to verapamil, which inhibits LTCC, increased the S/L ratio from 0.71 ± 0.16 to 0.94 ± 0.02 (*p* = 0.016) and decreased the CA values from 1.41 ± 0.24 to 0.49 ± 0.24 (*p* < 0.001, Figure 5b). Exposure to the SERCA blocker thapsigargin increased the S/L ratio from 0.78 ± 0.15 to 0.99 ± 0.01 (*p* = 0.016) and decreased the CA values from 1.25 ± 0.18 to 0.97 ± 0.19 (*p* < 0.001, Figure 5c). Exposure to ryanodine, which acts on ryanodine receptors, increased the S/L ratio from 0.64 ± 0.11 to 1.01 ± 0.02 (*p* = 0.001) and decreased the CA values from 1.30 ± 0.20 to 0.55 ± 0.33 (*p* < 0.001, Figure 5d). Exposure to the myosin inhibitor blebbistatin decreased the S/L ratio and CA values, from 0.65 ± 0.20 to 0.59 ± 0.20 (*p* < 0.001) and from 1.42 ± 0.23 to 1.04 ± 0.16 (*p* < 0.001, Figure 5e), respectively.

### 3.5 Effects of compounds on normal beating and C‐ALT in cardiac cell sheet tissues under spontaneous beating

**FIGURE 6:**
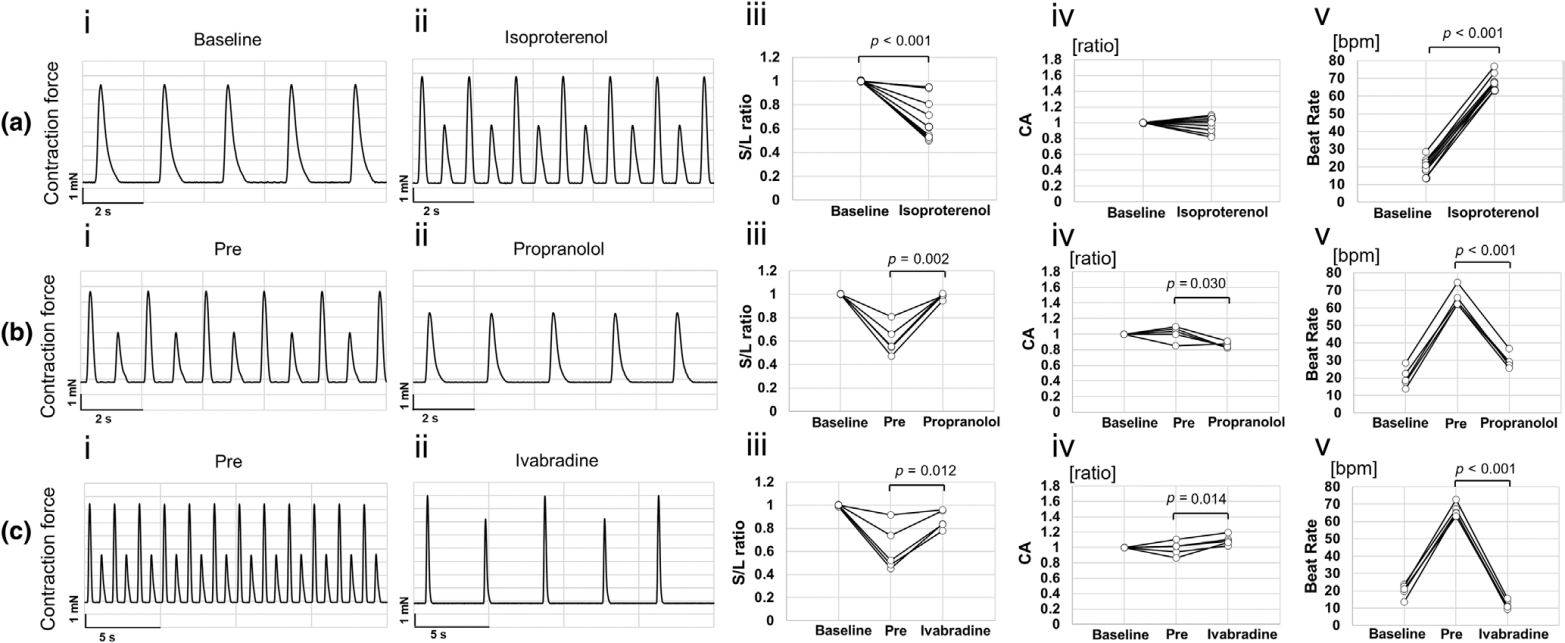
Effects of compounds on contractility of cardiac cell sheet tissues under spontaneous beating. (a–c) The typical contraction waveform, S/L ratio (each sample data), CA (contraction amplitude) value (each sample data), and beat rate (beats/min, each sample data) of cardiac cell sheet tissue, cultured under atmospheric condition using Medium 199 Hanks' salt medium, before (baseline) and after 30 min exposure to 1000 nM isoproterenol (S/L ratio [baseline]; mean ± S.D. = 1.00 ± 0.00, S/L ratio [isoproterenol]; 0.71 ± 0.19, CA [isoproterenol]; 0.97 ± 0.09, Beat Rate [baseline]; 20.2 ± 4.5, Beat Rate [isoproterenol]; 67.9 ± 4.2, *n* = 10), 1000 nM propranolol (S/L ratio [pre]; mean ± S.D. = 0.61 ± 0.13, S/L ratio [propranolol]; 0.98 ± 0.02, CA [pre]; 1.01 ± 0.10, CA [propranolol]; 0.86 ± 0.04, Beat Rate [pre]; 65.6 ± 5.2, Beat Rate [propranolol]; 29.5 ± 4.2, *n* = 5), and 300 nM ivabradine (S/L ratio [pre]; mean ± S.D. = 0.62 ± 0.20, S/L ratio [ivabradine]; 0.87 ± 0.08, CA [pre]; 0.99 ± 0.09, CA [ivabradine]; 1.09 ± 0.06, Beat Rate [pre]; 66.7 ± 3.7, Beat Rate [ivabradine]; 12.1 ± 2.4, *n* = 5), respectively. Propranolol and ivabradine were exposed to C‐ALT‐induced cardiac cell sheet tissue by exposure to isoproterenol. “Baseline” indicates no exposure to any compounds. “Pre” indicates that C‐ALT is induced by one micromolar of isoproterenol exposure. CA was normalized to baseline.

To determine whether the contractile response to isoproterenol differs between spontaneous beating and fixed beating rate, isoproterenol was exposed to cardiac cell sheet tissue under spontaneous beating conditions. As a result, the beat rate increased from 20.2 ± 4.5 to 67.9 ± 4.2 beats/min (*p* < 0.001), the S/L ratio decreased from 1.00 ± 0.00 to 0.71 ± 0.19 (*p* < 0.001), and the CA value did not change significantly (0.97 ± 0.09, *p* = 0.351, Figure 6a), compared to before isoproterenol exposure. Propranolol exposure in C‐ALT‐induced tissues under spontaneous beating decreased the beat rate from 65.6 ± 5.2 to 29.5 ± 4.2 beats/min (*p* < 0.001) and increased the S/L ratio from 0.61 ± 0.13 to 0.98 ± 0.02 (*p* = 0.002). The CA value changed slightly from 1.01 ± 0.10 to 0.86 ± 0.04 (*p* = 0.030, Figure 6b). Similarly, exposure to the HCN channel inhibitor ivabradine in C‐ALT‐induced tissues under spontaneous beating decreased the beat rate from 66.7 ± 3.7 to 12.1 ± 2.4 beats/min (*p* < 0.001) and increased the S/L ratio from 0.62 ± 0.20 to 0.87 ± 0.08 (*p* = 0.012) and CA values increased from 0.99 ± 0.09 to 1.09 ± 0.06 (*p* = 0.014, Figure 6c). Movie S5 shows C‐ALT‐induced cardiac cell sheet tissue after ivabradine exposure. The effects of each compound on C‐ALT are summarized in Table 3. In the table, “Disappeared” means one or more samples with the S/L ratio of 0.99 or greater after each compound exposure. “Suppressed” means an increase in the S/L ratio, but less than 0.99, after each compound exposure compared to before exposure.

### 3.6 Characteristic analysis of contraction waveforms of C‐ALT‐induced cardiac cell sheet tissues

**FIGURE 7:**
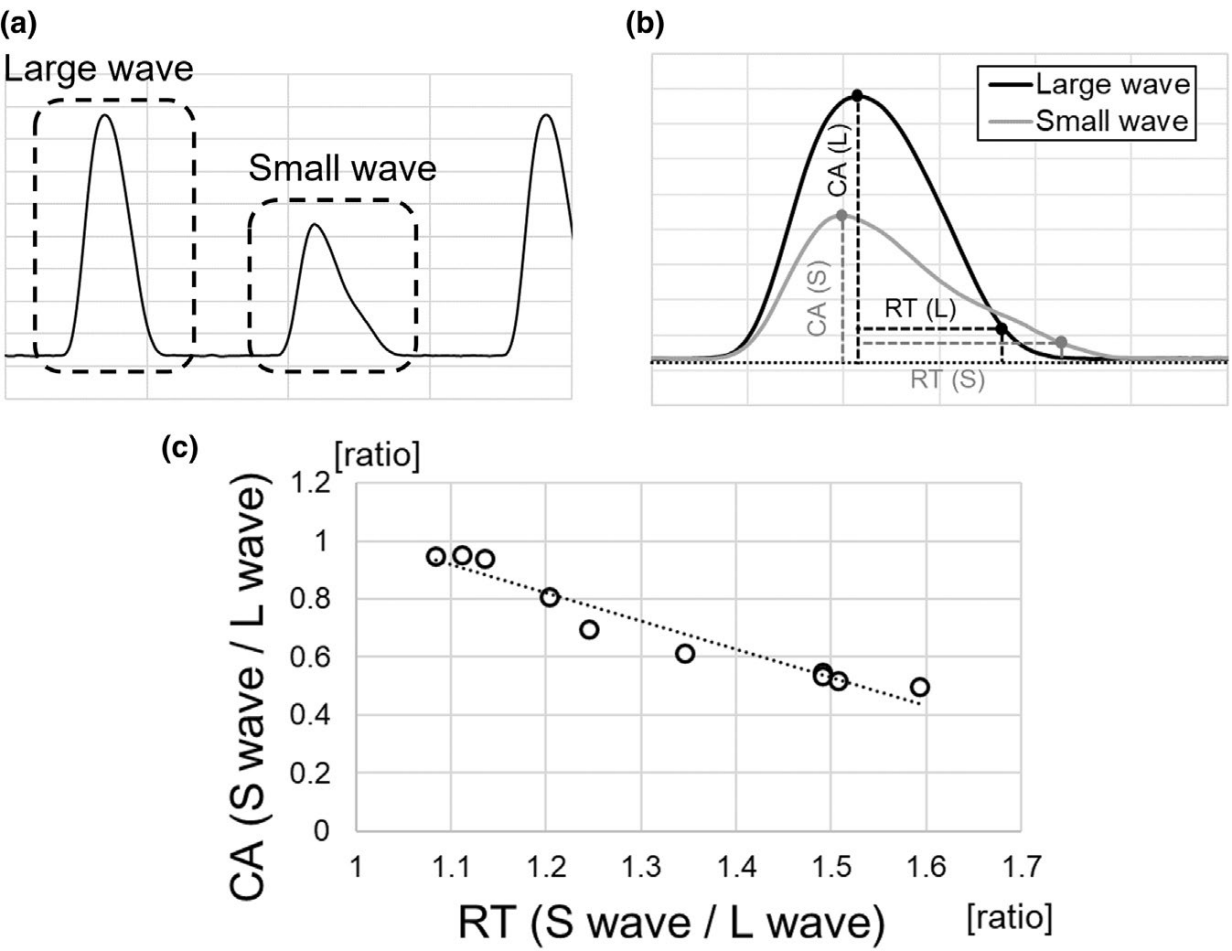
Contraction waveform analysis of C‐ALT in cardiac cell sheet tissues. (a) Typical contraction waveform in cardiac cell sheet tissues with C‐ALT under spontaneous beating conditions. (b) Overlapping of large and small contraction waveforms in cardiac cell sheet tissues with C‐ALT under spontaneous beating conditions. Black line indicates the large contraction waveform. Gray line indicates the small contraction waveform. CA (contraction amplitude) means the height from the baseline to the peak in waveform. RT (relaxation time) means the time taken for the tension to fall from 100% to 10% of the CA. (c) The relationship between CA and RT analyzed from large and small contraction waveforms of consecutive contractions in cardiac cell sheet tissues with C‐ALT under spontaneous beating conditions (*n* = 10). The vertical axis shows the ratio of the CA of two consecutive large and small waveforms. The horizontal axis shows the ratio of the RT of two consecutive large and small waveforms. The line shows the least‐square fitted lines using 10 plots (*y* = −0.975*x* + 1.992, *r* ^2^ = 0.938).

To analyze the contraction waveforms in 10 C‐ALT‐induced cardiac cell sheet tissues under spontaneous beating conditions shown in Figure 6a, the ratio of the wave heights for large and small contractions was plotted on the vertical axis, and the ratio of RTs for these contractions was plotted on the horizontal axis. From these, a linear approximation by the least‐squares method was calculated (Figure 7). As a result, the slopes of the least‐square fitted lines were −0.97 (*r* ^2^ = 0.938), indicating that the larger the difference in wave height between consecutive waveforms, the longer the relaxation time of small waveforms in cardiac cell sheet tissues with C‐ALT.

### 3.7 Relationship between characteristics of hiPSC‐CMs and C‐ALT induction

**FIGURE 8:**
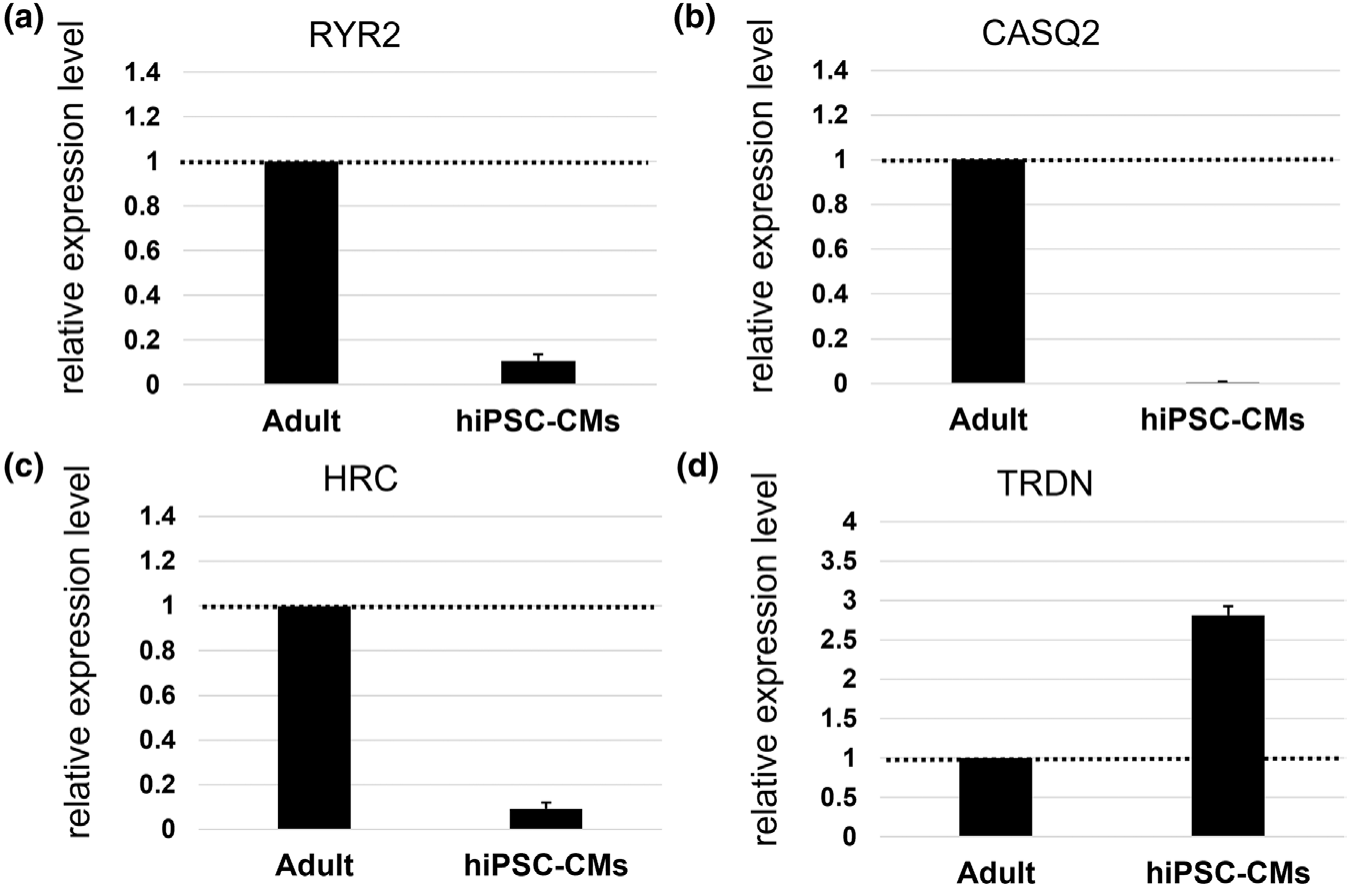
Gene expression of membrane proteins and calcium‐binding proteins in sarcoplasmic reticulum. (a–d) Quantitative analysis data of gene expression levels of RYR2, CASQ2, HRC, and TRDN using real‐time PCR. Gene expression levels for each data were normalized to the levels of adult (mean ± S.D.). Adult: Human primary adult heart.

The expression levels of *RYR2* (cardiac RyR gene responsible for Ca^2+^ release from the SR), *CASQ2* (calsequestrin2 gene), *HRC* (histidine‐rich calcium‐binding protein gene), and *TRDN* (triadin gene), which regulate RyR opening and closing, were examined in hiPSC‐CMs and human primary heart tissues using real‐time PCR. The results revealed that the expression levels of *RYR2*, *CASQ2*, and *HRC* in hiPSC‐CMs were less than 1 compared with those in human primary hearts, indicating the low expression of these genes in hiPSC‐CMs (95% CI: 0.037 to 0.179 for *RYR2*, 0.002 to 0.010 for *CASQ2*, and 0.058 to 0.126 for *HRC*). In contrast, the expression level of *TRDN* was higher than 1 compared with that in human primary hearts, confirming the high expression of this gene in hiPSC‐CMs (95% CI: 2.486 to 3.145 for *TRDN*) (Figure 8).

## 4 DISCUSSION

### 4.1 Relationship between contractility response to isoproterenol and culture environment of hiPSC‐CMs

In this study, C‐ALT was observed only in cardiac cell sheet tissues cultured under atmospheric conditions at pH 6.9 or 7.2 (CO_2_ concentration: approximately 0.04%, see Figure 2) when exposed to isoproterenol. On the contrary, C‐ALT was not observed in cardiac cell sheet tissues cultured under 5% CO_2_ conditions at pH 6.9 or 7.3. It is known that pH significantly affects cell properties and, as in vivo, the pH of the culture medium in vitro is mainly regulated by the HCO_3_^−^/CO_2_ system (H^+^ + HCO_3_^−^ ↔ H_2_CO_3_ ↔ CO_2_ + H_2_O) (Michl et al., 2019). In a 5% CO_2_ environment, CO_2_ dissolves in the medium, producing H^+^ and altering the pH. In this experiment, C‐ALT was not observed under both acidic (pH 6.9) and neutral (pH 7.2–7.3) conditions at 5% CO_2_ concentration. Although acidosis is known to induce C‐ALT, the results suggest that the pH change of the culture medium itself is unlikely to directly cause C‐ALT. On the contrary, C‐ALT was not observed in some of the tissues cultured at pH 6.9 under the atmospheric condition despite the fact that C‐ALT was observed in all tissues cultured at pH 7.2 under the atmospheric condition (see Figure 2b,c). These results suggest that pH may be related to the behavior of C‐ALT, and further studies are needed to clarify the relationship between pH and C‐ALT.

Bicarbonate ion (HCO_3_^−^), another molecule produced in a 5% CO_2_ environment, is one of the major anions in cardiomyocytes. HCO_3_^−^ may be closely related to normal contractile activity as it helps regulate ion transport in cardiomyocytes through transporters such as Cl^−^/HCO_3_^−^ exchanger and Na^+^/HCO_3_^−^ cotransporter (Alka & Casey, 2014; Wang, Chen, et al., 2014). This is also supported by reports showing that heart contractility decreases without HCO_3_^−^ (Fulop et al., 2003). Therefore, it is not surprising that C‐ALT, an abnormal contractile movement, was induced only in atmospheric environments where HCO_3_^−^ was not fully produced in the culture medium. Additionally, HCO_3_^−^ may directly influence contractility and Ca^2+^ handling; Watanabe et al. reported that receptors that regulate intracellular Ca^2+^ concentration in direct response to HCO_3_^−^ are expressed in brain mural cells (Jo‐Watanabe et al., 2024). Therefore, it is possible that cardiomyocytes also have mechanisms that directly sense HCO_3_^−^ concentrations. However, there are few studies on the direct effects of HCO_3_^−^ on contractility despite the known presence of several HCO_3_^−^ transporters in the heart. This study suggests that HCO_3_^−^ might directly influence the contractile response of cardiomyocytes to isoproterenol, but more research is needed to elucidate the mechanism.

### 4.2 Mechanism of C‐ALT induced by isoproterenol in hiPSC‐CMs

**FIGURE 9:**
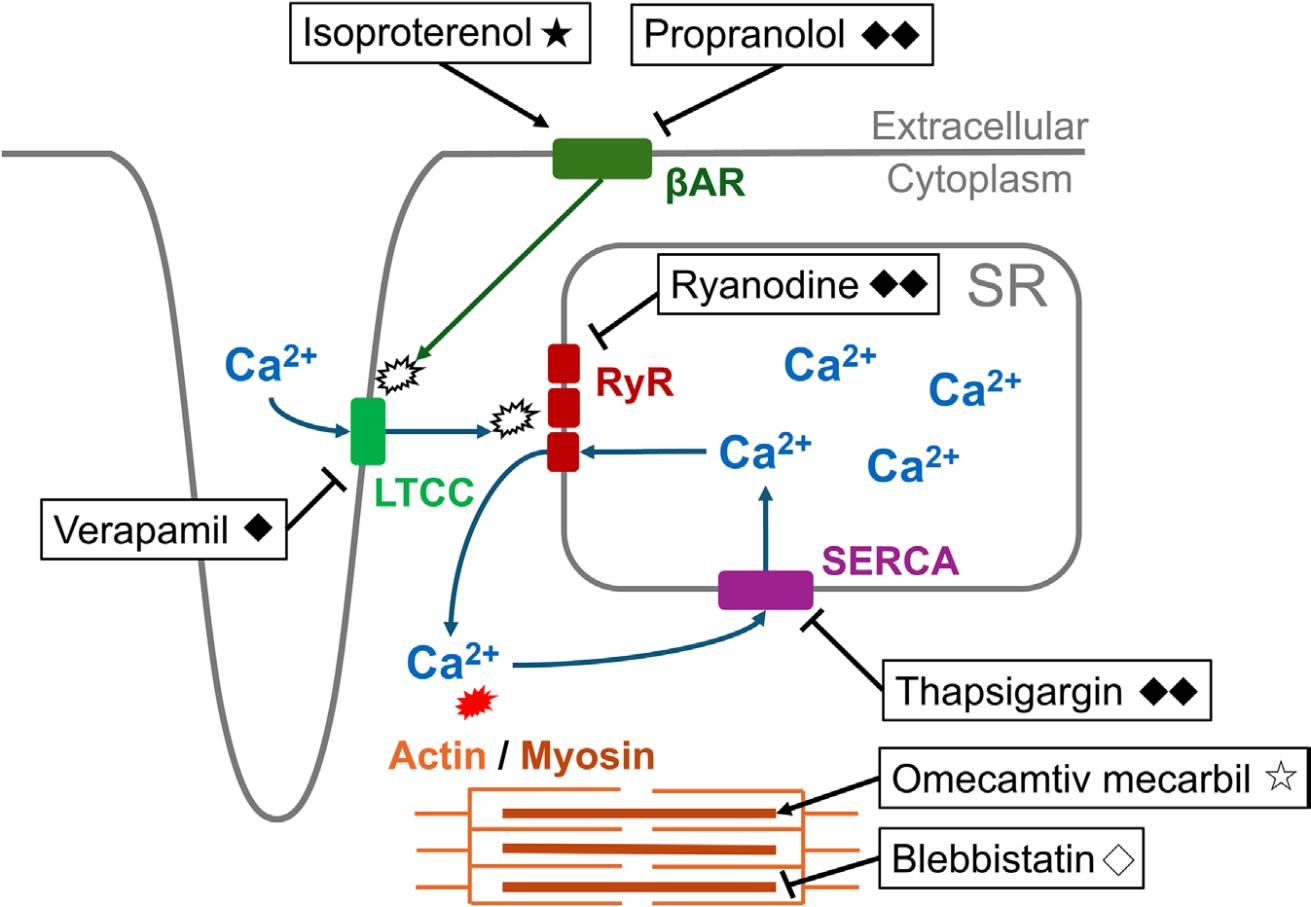
Schematic of Ca^2+^ dynamics in cardiomyocytes and compound mechanism of action. This figure illustrated the Ca^2+^ dynamics in cardiomyocytes and the effects of each compound observed in this study on C‐ALT. The meaning of each label and abbreviation are as follows: ◇, C‐ALT did not disappear or was suppressed; ◆, C‐ALT was suppressed; ◆◆, C‐ALT disappeared; ☆, C‐ALT was not induced; ★, C‐ALT was induced; LTCC, L‐type Ca^2+^ channel; RyR, ryanodine receptor; SERCA, sarco/endoplasmic reticulum Ca^2+^‐ATPase; SR, sarcoplasmic reticulum; βAR, beta adrenergic receptor.

Figure 9 shows the calcium kinetics in cardiomyocytes, the mechanism of action for each compound, and their effects on C‐ALT observed in this study. C‐ALT was induced by exposing cardiomyocytes to isoproterenol, a β‐adrenergic receptor stimulator. On the contrary, C‐ALT disappeared with exposure to the β‐adrenergic receptor blocker propranolol, suggesting that C‐ALT is induced by the β‐adrenergic receptor stimulation (Figures 2 and 5a). β‐adrenergic receptor stimulation of cardiomyocytes can either suppress or induce C‐ALT through changes in calcium dynamics (de Diego et al., 2008). Insufficient ATP relative to demand can promote C‐ALT induction in single cat atrial myocytes, with increased C‐ALT induction observed when mitochondrial activity is inhibited (Florea & Blatter, 2010). In this study, neither omecamtiv mecarbil, which boosts contractility by increasing the Ca^2+^ sensitivity of myosin, nor blebbistatin, which reduces contractility by inhibiting myosin, affected the induction or suppression of C‐ALT (Figures 4 and 5e). Although contractility changes would directly affect ATP demand, these results suggest that the C‐ALT in hiPSC‐CMs in this study is unrelated to changes in ATP demand.

It is well known that β‐adrenergic receptor stimulation in cardiomyocytes activates LTCC, SERCA, and RyR through protein kinase A activation. Since contractility is primarily controlled by the amount of Ca^2+^ released from the SR into the cytoplasm through the RyR, the activity of these channels that increases Ca^2+^ distribution induced by β‐adrenergic receptor stimulation will ultimately result in increased contractility. In this study, the LTCC blocker verapamil suppressed both contraction force and C‐ALT (Figure 5b). The data from omecamtiv mecarbil and blebbistatin rule out the possibility that C‐ALT is induced by a change in contractility without fluctuations in Ca^2+^ content. The results of verapamil indicate that the increase in cytosolic Ca^2+^ caused by LTCC activation by β‐adrenergic stimulation contributes to C‐ALT induction. On the contrary, C‐ALT was eliminated by the SERCA inhibitor thapsigargin and the RyR inhibitor ryanodine (Figure 5c,d). C‐ALT has been reported to occur due to a defect in the RyR, leading to fluctuations in the amount of Ca^2+^ released from the SR (Wang, Myles, et al., 2014). Therefore, the disappearance of C‐ALT following ryanodine exposure is not surprising and suggests that the C‐ALT observed in hiPSC‐CM is related to the status of the RyR. Additionally, the opening and closing of RyR are precisely regulated by the Ca^2+^ levels in the SR, which is controlled by SERCA, the enzyme responsible for its uptake (Bassani et al., 1995). Thus, the results of this study, where the SERCA inhibitor thapsigargin eliminated C‐ALT, suggest that increased Ca^2+^ load in the SR disrupts RyR regulation. In conclusion, the C‐ALT in hiPSC‐CMs exposed to isoproterenol may be due to abnormal RyR activity caused by excessive Ca^2+^ load in the SR due to β‐adrenergic receptor stimulation.

### 4.3 Relationship between the beating rate and C‐ALT in hiPSC‐CMs

C‐ALT was induced by isoproterenol exposure under both the 1.5‐Hz pacing rate and spontaneous beating in this study (Figure 6a). To further confirm the relationship between the beating rate and C‐ALT, tissues with C‐ALT under spontaneous beating were exposed to ivabradine, an HCN channel inhibitor, to reduce the beating rate. It suppressed C‐ALT but did not completely eliminate it (Figure 6c, Movie S5). It has been proposed that C‐ALT without changes in the amount of Ca^2+^ stored in the SR is caused by RyR refractoriness (Rovetti et al., 2010; Shkryl et al., 2012; Zhilin et al., 2016). RyRs are clustered on the SR membrane, and each RyR has its own refractory period. Under extremely high‐frequently beating, some RyRs do not recover in time for the next beat, resulting in fewer RyRs opening, less Ca^2+^ being released, and a reduced contractility one out of every two beats. Therefore, ivabradine suppressed C‐ALT, possibly because the reduced beat rate allowed time for recovery from the refractory period and increased the number of RyRs ready to open (Figure 6c). On the contrary, although ivabradine significantly reduced the beat rate from 66.7 ± 3.7 to 12.1 ± 2.4 bpm, it did not completely eliminate C‐ALT. Even in cardiac cell sheet tissue under C‐ALT, normal contraction (large contraction) was observed in one out of every two beats. If C‐ALT is due to RyR refractoriness, reducing the beating rate to less than half of that during C‐ALT will provide recovery time for RyR necessary to produce a normal beat (large contraction) and disappearance of C‐ALT.

In addition to C‐ALT caused by RyR refractoriness, other mechanisms have been reported, such as insufficient Ca^2+^ load in the SR (Díaz et al., 2004). However, the C‐ALT identified in this study showed a decrease in Ca^2+^ fluorescence intensity to baseline after each opening, indicating that inadequate SR Ca^2+^ recovery is unlikely to be the cause (see Figure 3). The contractile waveform showed that the contraction force returned to the same tension level after each relaxation, suggesting complete relaxation and that the SR Ca^2+^ load does not change due to insufficient Ca^2+^ recovery from the cytoplasm. Therefore, the finding that C‐ALT did not completely disappear even when the beat rate was reduced by ivabradine suggests that C‐ALT may not be solely due to RyR refractoriness. Alternatively, it suggests that RyR recovery requires factors other than time.

### 4.4 Characteristic of contraction waveforms of hiPSC‐CMs with C‐ALT

Analysis of the contraction waveforms of hiPSC‐CMs with C‐ALT revealed that the larger the difference in wave height values of two consecutive waveforms, the longer the RT of small waveforms (Figure 7). Exposing hiPSC‐CMs to compounds that target contraction‐related and calcium dynamics‐related proteins causes specific changes in the waveform. This suggests that analyzing the contraction waveform can reveal the active state of different proteins in cardiomyocytes (Hinata et al., 2022). It has been reported that relaxation in hiPSC‐CMs is primarily influenced by SERCA activity at the SR membrane (Jaferzadeh et al., 2023). Therefore, it can be inferred that SERCA activity is lower in the small waveform relaxation of C‐ALT than in the large waveform relaxation observed in this study. Since the SERCA activity is enhanced with higher cytoplasmic Ca^2+^ levels (Periasamy & Huke, 2001), it is not surprising that SERCA activity is low and RT was prolonged in small waveforms, where cytoplasmic Ca^2+^ is presumably lower than in large waveforms. However, nifedipine and verapamil, which lower intracytoplasmic Ca^2+^ levels in hiPSC‐CMs by inhibiting LTCC, do not significantly prolong RT (Mannhardt et al., 2017; Saleem et al., 2020). Therefore, it can be inferred that SERCA activity in cardiomyocytes during C‐ALT is influenced by factors other than the amount of Ca^2+^ in the cytosol. Besides the cytoplasmic Ca^2+^ concentration, SERCA activity is also influenced by the amount of Ca^2+^ in the SR immediately after contraction (Shannon et al., 2000): when SR Ca^2+^ levels are high, the rate of Ca^2+^ uptake by SERCA is limited. Therefore, the amount of Ca^2+^ remaining in the SR at the onset of relaxation in the small waveform of C‐ALT may be high, suppressing SERCA activity. This hypothesis is consistent with previous reports that C‐ALT is due to defective regulation of the RyR, resulting in inadequate Ca^2+^ release from the SR every two beats.

### 4.5 Characteristic of hiPSC‐CMs with C‐ALT

Despite extensive research on C‐ALT mechanisms, there are few reports of C‐ALT induction in vitro using human cells. This suggests that both the culture environment and the characteristics of hiPSC‐CMs may be involved in C‐ALT induction. As C‐ALT has been reported to be caused by defects in RyR regulation, we quantified the gene expression levels of three representative proteins—HRC, CSQ2, and TRDN—which are thought to be involved in regulating the opening and closing of RYR2, in hiPSC‐CMs and human primary adult heart. The results showed that two genes other than TRDN were less expressed in hiPSC‐CM than in human primary adult heart (Figure 8). HRC and CSQ2 are Ca^2+^‐binding proteins present in the SR lumen, and they are known to bind TRDN on the SR membrane during Ca^2+^ release from RYR2 (Arvanitis et al., 2011; Knollmann, 2009). HRC inhibits its action by interacting directly with SERCA at low SR Ca^2+^ concentrations, and when SR Ca^2+^ concentrations increase, it dissociates from SERCA and binds to TRDN, facilitating Ca^2+^ release from the RyR. The HRC is therefore thought to act as a Ca^2+^ sensor in the SR, regulating both Ca^2+^ recovery in the SR and its release into the cytosol (Arvanitis et al., 2011; Liu et al., 2015). CSQ2 binds to Ca^2+^ as its concentration in the SR rises and forms a complex with SR membrane proteins such as TRDN. This complex localizes Ca^2+^ near the RyR and promotes its release. Furthermore, HRC is known to increase RyR2 activity and promote diastolic recovery, whereas CSQ2 stabilizes RyR2 and makes it refractory (Liu et al., 2015). Although their exact roles in RyR regulation require further study, it is clear that HRC and CSQ2 are crucial for SR‐centered calcium dynamics. Indeed, it has been reported that mouse hearts with double knockout of HRC and CSQ2 expression showed normal cardiac ejection fraction and microstructure, but a significantly increased incidence of C‐ALT (Liu et al., 2015). Therefore, the low expression of HRC and CSQ2 in hiPSC‐CMs observed in this study may be involved in C‐ALT induction.

### 4.6 Study limitation

In this study, C‐ALT was successfully induced in hiPSC‐CMs in vitro. Analysis of the contractile response to compounds affecting calcium kinetics confirmed for the first time in human cells that changes in Ca^2+^ loading in the SR significantly influence C‐ALT induction. However, several limitations need to be addressed in further studies. One of these is the unclear relationship between atmospheric conditions, which are thought to induce C‐ALT, and the cell characteristics that may contribute to C‐ALT induction. Cultivating primary human heart fragments or cells in Tyrode's or Hanks' salt solutions under atmospheric conditions is not special, but there are few reports of C‐ALT induction. Therefore, both the culture environment and the cell characteristics may play crucial roles in C‐ALT induction. The lower expression of CSQ2 and HRC genes in hiPSC‐CMs compared to human primary adult heart observed in this study suggests potential instability in Ca^2+^ regulation in hiPSC‐CMs, but it is necessary to clarify how the culture environment influences Ca^2+^ regulation mechanism.

Another limitation in this study is that it does not consider the differences in physiological calcium dynamics and contractile mechanisms between hiPS‐CMs and the human heart. For example, the contributions of NCX and mitochondrial Ca^2+^ handling to contractility have been reported to differ between hiPSC‐CMs and human hearts (Zhang & Morad, 2020). While some of these differences in Ca^2+^ dynamics may be due to the immaturity of hiPSC‐CMs, C‐ALT is commonly observed in adult hearts with cardiac disease in clinical practice. C‐ALT development in hiPSC‐CMs might not be linked to their immaturity. Therefore, fully understanding the mechanism of C‐ALT requires more than just analyzing the contractile response and intracytoplasmic calcium imaging, and additional experiments from multiple perspectives that also consider the physiological differences between the hiPSC‐CMs and the human heart are needed.

## 5 CONCLUSION

In this study, C‐ALT was induced in vitro by β‐adrenergic receptor stimulation of hiPSC‐CMs cultured under atmospheric condition. In addition, C‐ALT mechanism observed in this study was inferred to be caused by an excessive increase in Ca^2+^ content in the SR, resulting in disrupted regulation of RyR opening and closing and Ca^2+^ release from the SR being insufficient every two beats. More interestingly, C‐ALT did not completely disappear even when the beating rate under spontaneous beating was reduced to a level considered sufficient for RyR recovery from refractoriness. This suggests that RyR refractoriness is influenced by factors other than time, or that there are factors besides RyR refractoriness that contribute to C‐ALT, and may provide new insights into C‐ALT mechanisms.

Although C‐ALT has been linked to severe heart failure and arrhythmias, the detailed developmental mechanism of C‐ALT in humans and its relationship to heart disease remain unclear, partly due to the lack of an in vitro model of C‐ALT using human cells. The C‐ALT model using hiPSC‐CMs generated in this study is expected to significantly advance our understanding of C‐ALT and aid in developing therapeutic strategies.

## AUTHOR CONTRIBUTIONS

Yuto Hinata: conceived and designed research, performed experiments, analyzed data, interpreted results of experiments, prepared figures, drafted manuscript, edited and revised manuscript. Daisuke Sasaki and Katsuhisa Matsuura: conceived and designed research, interpreted results of experiments, edited and revised manuscript. Tatsuya Shimizu: conceived and designed research, interpreted results of experiments, edited and revised manuscript, approved final version of manuscript.

## FUNDING INFORMATION

This study is supported in part by the Agency for Medical Research and Development (AMED, grant number: 22mk0101189h0102, 23mk0101189h0103).

## CONFLICT OF INTEREST STATEMENT

Yuto Hinata is an employee of Nihon Kohden Corporation. Tatsuya Shimizu is a stakeholder in CellSeed Inc. Tokyo Women's Medical University receives research funding from CellSeed Inc. and Nihon Kohden Corporation. Katsuhisa Matsuura and Tatsuya Shimizu are inventors of bioreactor systems.

## ETHICS STATEMENT

None.

## Supporting information


Table S1. The passage number of iPS cells and the n number of each experiment

Movie S1. Calcium dynamics of hiPSC‐CMs (before isoproterenol exposure)

Movie S2. Calcium dynamics of hiPSC‐CMs (after isoproterenol exposure)

Movie S3. Calcium dynamics of cardiac cell sheet tissue (before isoproterenol exposure)

Movie S4. Calcium dynamics of cardiac cell sheet tissue (after isoproterenol exposure)

Movie S5. C‐ALT‐induced cardiac cell sheet tissue (after ivabradine exposure)

## Data Availability

The data that support the finding of this study are available from the corresponding authors upon reasonable request.

## References

[phy270152-bib-0001] Alka, K. , & Casey, J. R. (2014). Bicarbonate transport in health and disease. IUBMB Life, 66(9), 596–615. 10.1002/iub.1315 25270914

[phy270152-bib-0002] Arai, K. , Murata, D. , ShokoTakao, A. N. , Itoh, M. , Kitsuka, T. , & Nakayama, K. (2020). Drug response analysis for scaffold‐free cardiac constructs fabricated using bio‐3D printer. Scientific Reports, 10, 8972. 10.1038/s41598-020-65681-y 32487993 PMC7265390

[phy270152-bib-0003] Arvanitis, D. A. , Vafiadaki, E. , Sanoudou, D. , & Kranias, E. G. (2011). Histidine‐rich calcium binding protein: The new regulator of sarcoplasmic reticulum calcium cycling. Journal of Molecular and Cellular Cardiology, 50, 43–49. 10.1016/j.yjmcc.2010.08.021 20807542 PMC3018531

[phy270152-bib-0004] Bassani, J. W. M. , Yuan, W. , & Bers, D. M. (1995). Fractional SR Ca release is regulated by trigger Ca and SR Ca content in cardiac myocytes. American Journal of Physiology. Cell Physiology, 268, C1313–C1319. 10.1152/ajpcell.1995.268.5.C1313 7762626

[phy270152-bib-0005] de Diego, C. , Chen, F. , Xie, L.‐H. , Dave, A. S. , Thu, M. , Rongey, C. , Weiss, J. N. , & Valderra'bano, M. (2008). Cardiac alternans in embryonic mouse ventricles. American Journal of Physiology. Heart and Circulatory Physiology, 294, H433–H440. 10.1152/ajpheart.01165.2007 18024542

[phy270152-bib-0006] Díaz, M. E. , O'Neill, S. C. , & Eisner, D. A. (2004). Sarcoplasmic reticulum calcium content fluctuation is the key to cardiac alternans. Circulation Research, 94, 650–656. 10.1161/01.RES.0000119923.64774.72 14752033

[phy270152-bib-0007] Edwards, J. N. , & Blatter, L. A. (2014). Cardiac alternans and intracellular calcium cycling. Clinical and Experimental Pharmacology & Physiology, 41, 524–532. 10.1111/1440-1681.12231 25040398 PMC4122248

[phy270152-bib-0008] Florea, S. M. , & Blatter, L. A. (2010). The role of mitochondria for the regulation of cardiac alternans. Frontiers in Physiology, 1, 141. 10.3389/fphys.2010.00141 21423381 PMC3059961

[phy270152-bib-0009] Florea, S. M. , & Blatter, L. A. (2012). Regulation of cardiac alternans by β‐adrenergic signaling pathways. American Journal of Physiology. Heart and Circulatory Physiology, 303, H1047–H1056. 10.1152/ajpheart.00384.2012 22904161 PMC3469647

[phy270152-bib-0010] Fulop, L. , Szigeti, G. , Magyar, J. , Szentandrassy, N. , Ivanics, T. , Miklos, Z. , Ligeti, L. , Kovacs, A. , Szenasi, G. , Csernoch, L. , Nanasi, P. P. , & Banyasz, T. (2003). Differences in electrophysiological and contractile properties of mammalian cardiac tissues bathed in bicarbonate – And HEPES‐buffered solutions. Acta Physiologica Scandinavica, 178, 11–18. 10.1046/j.1365-201X.2003.01114.x 12713510

[phy270152-bib-0011] Haghighi, K. , Kolokathis, F. , Pater, L. , Lynch, R. A. , Asahi, M. , Gramolini, A. O. , Fan, G.‐C. , Tsiapras, D. , Hahn, H. S. , Adamopoulos, S. , Liggett, S. B. , Dorn, G. W., 2nd , MacLennan, D. H. , Kremastinos, D. T. , & Kranias, E. G. (2003). Human phospholamban null results in lethal dilated cardiomyopathy revealing a critical difference between mouse and human. Journal of Clinical Investigation, 111, 869–876. 10.1172/JCI200317892 12639993 PMC153772

[phy270152-bib-0012] Haraguchi, Y. , Kagawa, Y. , Hasegawa, A. , Kubo, H. , & Shimizu, T. (2018). Rapid fabrication of detachable three‐dimensional tissues by layering of cell sheets with heating centrifuge. Biotechnology Progress, 34, 692–701. 10.1002/btpr.2612 29345093

[phy270152-bib-0013] Hinata, Y. , Kagawa, Y. , Kubo, H. , Kato, E. , Baba, A. , Sasaki, D. , Matsuura, K. , Sawada, K. , & Shimizu, T. (2022). Importance of beating rate control for the analysis of drug effects on contractility in human induced pluripotent stem cell‐derived cardiomyocytes. Journal of Pharmacological and Toxicological Methods, 118, 107228. 10.1016/j.vascn.2022.107228 36273536

[phy270152-bib-0014] Jaferzadeh, K. , Rappaz, B. , Kim, Y. , Kim, B.‐K. , Moon, I. , Marquet, P. , & Turcatti, G. (2023). Automated dual‐mode cell monitoring to simultaneously explore calcium dynamics and contraction−relaxation kinetics within drug‐treated stem cell‐derived cardiomyocytes. ACS Sensors, 8, 2533–2542. 10.1021/acssensors.3c00073 37335579

[phy270152-bib-0015] Jordan, P. N. , & Christini, D. J. (2007). Characterizing the contribution of voltage‐ and calcium‐dependent coupling to action potential stability: Implications for repolarization alternans. American Journal of Physiology. Heart and Circulatory Physiology, 293, H2109–H2118. 10.1152/ajpheart.00609.2007 17586611

[phy270152-bib-0016] Jo‐Watanabe, A. , Inaba, T. , Osada, T. , Hashimoto, R. , Nishizawa, T. , Okuno, T. , Ihara, S. , Touhara, K. , Hattori, N. , Oh‐Hora, M. , Nureki, O. , & Yokomizo, T. (2024). Bicarbonate signaling via G protein‐coupled receptor regulates ischaemia‐reperfusion injury. Nature Communications, 15, 1530. 10.1038/s41467-024-45579-3 PMC10899177 38413581

[phy270152-bib-0017] Knollmann, B. C. (2009). New roles of calsequestrin and triadin in cardiac muscle. The Journal of Physiology, 587, 3081–3087. 10.1113/jphysiol.2009.172098 19451205 PMC2727016

[phy270152-bib-0018] Kodama, M. , Kato, K. , Hirono, S. , Okura, Y. , Hanawa, H. , Ito, M. , Fuse, K. , Shiono, T. , Watanabe, K. , & Aizawa, Y. (2001). Mechanical alternans in patients with chronic heart failure. Journal of Cardiac Failure, 7, 138–145. 10.1054/jcaf.2001.24122 11420765

[phy270152-bib-0019] Kulkarni, K. , Merchant, F. M. , Kassab, M. B. , Sana, F. , Moazzami, K. , Sayadi, O. , Singh, J. P. , Kevin Heist, E. , & Armoundas, A. A. (2019). Cardiac alternans: Mechanisms and clinical utility in arrhythmia prevention. Journal of the American Heart Association, 8, e013750. 10.1161/JAHA.119.013750 31617437 PMC6898836

[phy270152-bib-0020] Lab, M. J. , & Lee, J. A. (1990). Changes in intracellular calcium during mechanical alternans in isolated ferret ventricular muscle. Circulation Research, 66, 585–595. 10.1161/01.RES.66.3.585 2306800

[phy270152-bib-0021] Liu, B. , Ho, H.‐T. , Brunello, L. , Unudurthi, S. D. , Lou, Q. , Belevych, A. E. , Qian, L. , Kim, D. H. , Cho, C. , Janssen, P. M. L. , Hund, T. J. , Knollmann, B. C. , Kranias, E. G. , & Gyo¨rke, S'n. (2015). Ablation of HRC alleviates cardiac arrhythmia and improves abnormal Ca handling in CASQ2 knockout mice prone to CPVT. Cardiovascular Research, 108, 299–311. 10.1093/cvr/cvv222 26410369 PMC4614688

[phy270152-bib-0022] Mannhardt, I. , Breckwoldt, K. , Letuffe‐Brenie're, D. , Schaaf, S. , Schulz, H. , Neuber, C. , Benzin, A. , Werner, T. , Eder, A. , Schulze, T. , Klampe, B. , Christ, T. , Hirt, M. N. , Huebner, N. , Moretti, A. , Eschenhagen, T. , & Hansen, A. (2016). Human engineered heart tissue: Analysis of contractile force. Stem Cell Reports, 7, 29–42. 10.1016/j.stemcr.2016.04.011 27211213 PMC4944531

[phy270152-bib-0023] Mannhardt, I. , Eder, A. , Dumotier, B. , Prondzynski, M. , Kramer, E. , Traebert, M. , So¨hren, K.‐D. , Flenner, F. , Stathopoulou, K. , Lemoine, M. D. , Carrier, L. , Christ, T. , Eschenhagen, T. , & Hansen, A. (2017). Blinded contractility analysis in hiPSC‐cardiomyocytes in engineered heart tissue format: Comparison with human atrial Ttrabeculae. Toxicological Sciences, 158(1), 164–175. 10.1093/toxsci/kfx081 28453742 PMC5837217

[phy270152-bib-0024] Mannhardt, I. , Saleem, U. , Mosqueira, D. , Loos, M. F. , Ba¨rbel, M. , Ulmer, M. D. , Lemoine, C. L. , Caroline, A'e. , de Korte, T. , Vlaming, M. L. H. , Harris, K. , Clements, P. , Denning, C. , Hansen, A. , & Eschenhagen, T. (2020). Comparison of 10 control hPSC lines for drug screening in an engineered heart tissue format. Stem Cell Reports, 15, 983–998. 10.1016/j.stemcr.2020.09.002 33053362 PMC7561618

[phy270152-bib-0025] Matsuura, K. , Seta, H. , Haraguchi, Y. , Alsayegh, K. , Sekine, H. , Shimizu, T. , Hagiwara, N. , Yamazaki, K. , & Okano, T. (2016). TRPV‐1‐mediated elimination of residual iPS cells in bioengineered cardiac cell sheet tissues. Scientific Reports, 6, 21747. 10.1038/srep21747 26888607 PMC4757885

[phy270152-bib-0026] Matsuura, K. , Wada, M. , Shimizu, T. , Haraguchi, Y. , Sato, F. , Sugiyama, K. , Konishi, K. , Shiba, Y. , Ichikawa, H. , Tachibana, A. , Ikeda, U. , Yamato, M. , Hagiwara, N. , & Okano, T. (2012). Creation of human cardiac cell sheets using pluripotent stem cells. Biochemical and Biophysical Research Communications, 425, 321–327. 10.1016/j.bbrc.2012.07.089 22842572

[phy270152-bib-0027] Mcgaughey, M. D. , Lowell Maughan, W. , Sunagawa, K. , & Sagawa, K. (1985). Alternating contractility in pulsus alternans studied in the isolated canine heart. Circulation, 71, 357–362. 10.1161/01.CIR.71.2.357 3965175

[phy270152-bib-0028] Michl, J. , Park, K. C. , & Swietach, P. (2019). Evidence‐based guidelines for controlling pH in mammalian live‐cell culture systems. Communications Biology, 2, 144. 10.1038/s42003-019-0393-7 31044169 PMC6486606

[phy270152-bib-0029] Orchard, C. H. , Macall, E. , Kirby, M. S. , & Boyett, M. R. (1991). Mechanical alternans during acidosis in ferret heart muscle. Circulation Research, 68, 69–76. 10.1161/01.RES.68.1.69 1984873

[phy270152-bib-0030] Pang, L. , Sager, P. , Yang, X. , Shi, H. , Sannajust, F. , Brock, M. , Wu, J. C. , Abi‐Gerges, N. , Lyn‐Cook, B. , Berridge, B. R. , & Stockbridge, N. (2019). Workshop report: FDA workshop on improving cardiotoxicity assessment with human‐relevant platforms. Circulation Research, 125(9), 855–867. 10.1161/CIRCRESAHA.119.315378 31600125 PMC6788760

[phy270152-bib-0031] Periasamy, M. , & Huke, S. (2001). SERCA pump level is a critical determinant of Ca^2+^ homeostasis and cardiac contractility. Journal of Molecular and Cellular Cardiology, 33, 1053–1063. 10.1006/jmcc.2001.1366 11444913

[phy270152-bib-0032] Picht, E. , DeSantiago, J. , Blatter, L. A. , & Bers, D. M. (2006). Cardiac alternans do not rely on diastolic sarcoplasmic reticulum calcium content fluctuations. Circulation Research, 99, 740–748. 10.1161/01.RES.0000244002.88813.91 16946134

[phy270152-bib-0033] Rovetti, R. , Cui, X. , Garfinkel, A. , Weiss, J. N. , & Zhilin, Q. (2010). Spark‐induced sparks as a mechanism of intracellular calcium alternans in cardiac myocytes. Circulation Research, 106, 1582–1591. 10.1161/CIRCRESAHA.109.213975 20378857 PMC2893409

[phy270152-bib-0034] Saleem, U. , van Meer, B. J. , Katili, P. A. , Mohd, N. A. N. , Yusof, I. M. , Garcia, A. K. , Tertoolen, L. , de Korte, T. , Vlaming, M. L. H. , McGlynn, K. , Nebel, J. , Bahinski, A. , Harris, K. , Rossman, E. , Xiaoping, X. , Burton, F. L. , Smith, G. L. , Clements, P. , Mummery, C. L. , … Denning, C. (2020). Blinded, multicenter evaluation of drug‐induced changes in contractility using human‐induced pluripotent stem cell‐derived cardiomyocytes. Toxicological Sciences, 176, 103–123. 10.1093/toxsci/kfaa058 32421822 PMC7357169

[phy270152-bib-0035] Sasaki, D. , Matsuura, K. , Seta, H. , Haraguchi, Y. , Okano, T. , & Shimizu, T. (2018). Contractile force measurement of human induced pluripotent stem cell‐derived cardiac cell sheet‐tissue. PLoS One, 13, e0198026. 10.1371/journal.pone.0198026 29791489 PMC5965888

[phy270152-bib-0036] Shannon, T. R. , Ginsburg, K. S. , & Bers, D. M. (2000). Reverse mode of the sarcoplasmic reticulum calcium pump and load‐dependent cytosolic calcium decline in voltage‐clamped cardiac ventricular myocytes. Biophysical Journal, 78, 322–333. 10620296 10.1016/S0006-3495(00)76595-7 PMC1300640

[phy270152-bib-0037] Shkryl, V. M. , Maxwell, J. T. , Domeier, T. L. , & Blatter, L. A. (2012). Refractoriness of sarcoplasmic reticulum Ca^2+^ release determines Ca^2+^ alternans in atrial myocytes. American Journal of Physiology. Heart and Circulatory Physiology, 302, H2310–H2320. 10.1152/ajpheart.00079.2012 22467301 PMC3378301

[phy270152-bib-0038] Takahashi, K. , Tanabe, K. , Ohnuki, M. , Narita, M. , Ichisaka, T. , Tomoda, K. , & Yamanaka, S. (2007). Induction of pluripotent stem cells from adult human fibroblasts by defined factors. Cell, 131(5), 861–872. 10.1016/j.cell.2007.11.019 18035408

[phy270152-bib-0039] Walker, M. L. , & Rosenbaum, D. S. (2003). Repolarization alternans: Implications for the mechanism and prevention of sudden cardiac death. Cardiovascular Research, 57, 599–614. 10.1016/S0008-6363(02)00737-X 12618222

[phy270152-bib-0040] Wan, X. , Cutler, M. , Song, Z. , Karma, A. , Matsuda, T. , Baba, A. , & Rosenbaum, D. S. (2012). New experimental evidence for mechanism of arrhythmogenic membrane potential alternans based on balance of electrogenic I_NCX_/I_Ca_ currents. Heart Rhythm, 9, 1698–1705. 10.1016/j.hrthm.2012.06.031 22721857 PMC3459151

[phy270152-bib-0041] Wang, H.‐S. , Chen, Y. , Vairamani, K. , & Shull, G. E. (2014). Critical role of bicarbonate and bicarbonate transporters in cardiac function. World Journal of Biological Chemistry, 5(3), 334–345. 10.4331/wjbc.v5.i3.334 25225601 PMC4160527

[phy270152-bib-0042] Wang, L. , Myles, R. C. , De Jesus, N. M. , Ohlendorf, A. K. P. , Bers, D. M. , & Ripplinger, C. M. (2014). Optical mapping of sarcoplasmic reticulum Ca^2+^ in the intact heart. Circulation Research, 114, 1410–1421. 10.1161/CIRCRESAHA.114.302505 24568740 PMC4000583

[phy270152-bib-0043] Xie, L.‐H. , Sato, D. , Garfinkel, A. , Zhilin, Q. , & Weiss, J. N. (2008). Intracellular Ca alternans: Coordinated regulation by sarcoplasmic reticulum release, uptake, and leak. Biophysical Journal, 95, 3100–3110. 10.1529/biophysj.108.130955 18539635 PMC2527258

[phy270152-bib-0044] Zhang, X.‐h. , & Morad, M. (2020). Ca^2+^ signaling of human pluripotent stem cells‐derived cardiomyocytes as compared to adult mammalian cardiomyocytes. Cell Calcium, 90, 102244. 10.1016/j.ceca.2020.102244 32585508 PMC7483365

[phy270152-bib-0045] Zhilin, Q. , Liu, M. B. , & Nivala, M. (2016). A unified theory of calcium alternans in ventricular myocytes. Scientific Reports, 6, 35625. 10.1038/srep35625 27762397 PMC5071909

[phy270152-bib-0046] Zhilin, Q. , & Weiss, J. N. (2023). Cardiac alternans: From bedside to bench and back. Circulation Research, 132, 127–149. 10.1161/CIRCRESAHA.122.321668 36603066 PMC9829045

