# Supplementary material for: Induction of cardiac alternans in human iPS‐derived cardiomyocytes through β‐adrenergic receptor stimulation
Source: Physiol Rep. 2024 Dec 23;12(24):e70152. doi: 10.14814/phy2.70152 (PMC11666346; doi:10.14814/phy2.70152)
Supplement: Supplementary file 1 — Table S1. The passage number of iPS cells and the n number of each experiment [file PHY2-12-e70152-s002.docx]

**Supplementary Table 1. The passage number of iPS cells and the n number of each experiment**

The numbers shown in parentheses indicate the number of samples that were tested with the tissues used experiment shown in Fig. 2B or Fig. 6A.
